# Characterization of mutants deficient in N-terminal phosphorylation of the chloroplast ATP synthase subunit β

**DOI:** 10.1093/plphys/kiad013

**Published:** 2023-01-13

**Authors:** Deserah D Strand, Daniel Karcher, Stephanie Ruf, Anne Schadach, Mark A Schöttler, Omar Sandoval-Ibañez, David Hall, David M Kramer, Ralph Bock

**Affiliations:** Max-Planck-Institut für Molecular Pflanzenphysiologie, Am Mühlenberg 1, D-14476 Potsdam-Golm, Germany; Max-Planck-Institut für Molecular Pflanzenphysiologie, Am Mühlenberg 1, D-14476 Potsdam-Golm, Germany; Max-Planck-Institut für Molecular Pflanzenphysiologie, Am Mühlenberg 1, D-14476 Potsdam-Golm, Germany; Max-Planck-Institut für Molecular Pflanzenphysiologie, Am Mühlenberg 1, D-14476 Potsdam-Golm, Germany; Max-Planck-Institut für Molecular Pflanzenphysiologie, Am Mühlenberg 1, D-14476 Potsdam-Golm, Germany; Max-Planck-Institut für Molecular Pflanzenphysiologie, Am Mühlenberg 1, D-14476 Potsdam-Golm, Germany; DOE Plant Research Laboratory, Michigan State University, 612 Wilson Rd 106, East Lansing, Michigan, 48824, USA; DOE Plant Research Laboratory, Michigan State University, 612 Wilson Rd 106, East Lansing, Michigan, 48824, USA; Max-Planck-Institut für Molecular Pflanzenphysiologie, Am Mühlenberg 1, D-14476 Potsdam-Golm, Germany

## Abstract

Understanding the regulation of photosynthetic light harvesting and electron transfer is of great importance to efforts to improve the ability of the electron transport chain to supply downstream metabolism. A central regulator of the electron transport chain is ATP synthase, the molecular motor that harnesses the chemiosmotic potential generated from proton-coupled electron transport to synthesize ATP. ATP synthase is regulated both thermodynamically and post-translationally, with proposed phosphorylation sites on multiple subunits. In this study we focused on two N-terminal serines on the catalytic subunit β in tobacco (*Nicotiana tabacum*), previously proposed to be important for dark inactivation of the complex to avoid ATP hydrolysis at night. Here we show that there is no clear role for phosphorylation in the dark inactivation of ATP synthase. Instead, mutation of one of the two phosphorylated serine residues to aspartate to mimic constitutive phosphorylation strongly decreased ATP synthase abundance. We propose that the loss of N-terminal phosphorylation of ATPβ may be involved in proper ATP synthase accumulation during complex assembly.

## Teaser

N-terminal phosphorylation of ATP synthase subunit β interferes with complex accumulation during assembly.

## Introduction

Photosynthesis provides the starting material for nearly all life on earth. There is currently a drive to improve photosynthesis to meet food demands of a growing global population and make renewable biofuels to combat climate change. Understanding photosynthesis and how it is regulated is important if we want to modify these reactions to increase plant growth or increase flexibility to respond to stress conditions (Ort et al., 2015).

The so-called “light reactions” of photosynthesis are comprised of the thylakoid embedded electron transport chain that feeds the Calvin-Benson-Bassham cycle with the ATP and NADPH required for CO_2_ fixation. The central regulator of the thylakoid reactions of photosynthesis is the ATP synthase (discussed in (Kramer et al., 2004)). ATP synthase is a molecular motor comprised of a membrane associate CF_o_ subcomplex and a soluble CF_1_ subcomplex that work together to convert protonmotive force (*pmf*), generated by proton-coupled electron transfer, into phosphorylation potential in the form of ATP (Hahn et al., 2018). The ΔpH component of *pmf* activates pH-dependent feedback regulation of light harvesting (i.e. exciton quenching, q_E_), the largest component of non-photochemical quenching (NPQ) and lowers the rate constant for deprotonation of plastoquinol at the cytochrome *b_6_f* (*bf*) complex Q_o_ site (photosynthetic control) (Eberhard et al., 2008). In high light, *q_E_* and photosynthetic control protect photosystem II and I, respectively, from photodamage (Li et al., 2002; Suorsa et al., 2012). Changing the activation state of the ATP synthase has a rapid direct effect on these photoprotective processes (Kanazawa and Kramer, 2002; Takizawa et al., 2008; Zhang et al., 2009), and the understanding of ATP synthase regulation is important for our understanding of the regulation of photosynthesis as a whole.

In response to long-term changes in the metabolic demand for ATP and NADPH during leaf ontogenesis or in response to abiotic stresses, the content of ATP synthase is strongly regulated and usually changes in parallel with linear electron transport capacity and the abundance of the *bf* complex [reviewed in (Schöttler et al., 2015)]. However, because these changes in ATP synthase content occur over many days to weeks, they are unlikely to play a role in short-term adjustments of ATP synthase activity during the day. Here, indications exist that two pools of ATP synthase usually exist, one active, and the other inactive. In tobacco mutants, ATP synthase content could be reduced by more than 50%, without clear effects on ATP synthase activity, likely because an inactive enzyme pool was (re)-activated (Rott et al., 2011). However, the underlying regulation mechanisms need further elucidation: ATP synthase is regulated by substrate availability, protein-protein interactions, and multiple post-translational modifications. While it is unlikely that, in the light, thermodynamics would favor ATP hydrolysis, metabolic changes may still contribute to altered kinetics under conditions where substrate is limited (Kanazawa and Kramer, 2002; Takizawa et al., 2008). In the dark, the ATPγ subunit of ATP synthase (encoded by the *ATPC1* gene in the nuclear genome) is oxidized, and leads to a decrease in the rate constant of proton efflux (Wu et al., 2007; Kohzuma et al., 2013).

An additional post-translational modification proposed to modulate ATP synthase activity during light to dark transitions and substrate limitation is phosphorylation (Bunney et al., 2001; Takizawa et al., 2008; Reiland et al., 2009). While there have been multiple proposed phosphorylation sites on ATP synthase, two serine residues in the N-terminal domain of the chloroplast-encoded CF_1_-β subunit (encoded by *atpB*) have raised particular interest, because they were shown to be phosphorylated at the end of the night (del Riego et al., 2006; Reiland et al., 2009). Due to the timing of the phosphorylation, it was proposed that these residues are specifically involved in the inactivation of ATP synthase in the dark to avoid ATP hydrolysis. In this work, we set out to test this hypothesis by mutating these two residues (serine 8 and serine 13) to either an alanine, to eliminate the phosphorylation site, or an aspartic acid, to mimic constitutive phosphorylation. To assess the function of each of these residues in ATP synthase dark inactivation, we used chloroplast transformation to generate a set of mutants with all possible combinations of phosphorylation states.

## Results

### Generation of transplastomic tobacco plants with mutated phosphorylation sites in the *atpB* gene

To test the hypothesis that phosphorylation of the N-terminus of ATPβ is involved in regulation of ATP synthase, we introduced mutations either abolishing the phosphorylation site (S-to-A) or mimicking a phosphorylated residue (S-to-D). To achieve this, we used co-transformation of 9 plasmids: 8 plasmids containing our mutations of interest in all combinations, and one plasmid with the *aadA* selectable marker gene cassette. This allowed us to identify plants resistant to spectinomycin (conferred by the *aadA* gene; (Svab and Maliga, 1993; Bock, 2015)), and then further screen these transplastomic plants for the presence of any of the desired mutations, allowing for isolation of multiple mutations within the same transformation experiment. After biolistic bombardment and selection, the first round of plant regenerants were screened by PCR and sequencing of the *atpB* gene (encoding ATPβ). Plants that contained the desired mutation were sub-cultured further, and the second round of regenerated plants were screened again by PCR and DNA sequencing. Plants appearing to have a single peak in the Sanger sequencing chromatogram were further analyzed by Southern blotting for homoplasmic incorporation of the selection cassette. Plants that appeared to be homoplasmic for both the *atpB* mutation and the co-transformed selection cassette were transferred to the greenhouse for seed production.

**Figure 1:**
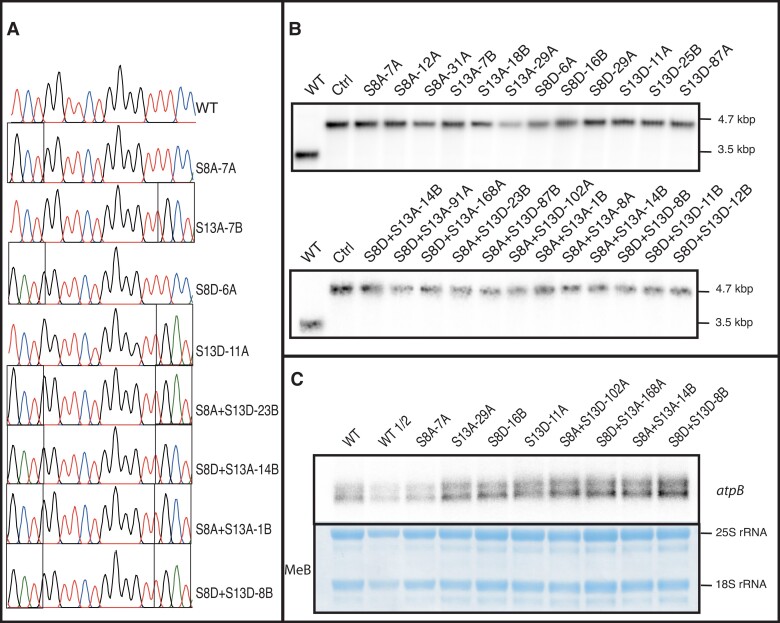
Molecular characterization of ATPβ mutants. A, Sanger chromatograms from DNA of ∼20 pooled seedlings resistant to spectinomycin. Black boxes indicate codons harboring mutations; B, Southern blot of DNA from A using a probe against *psaB*; C, Northern blot of RNA from homoplasmic representative plants determined from (A) and (B). MeB: Methylene blue staining of membrane. WT: *N. tabacum* “Petit Havana”.

To determine homoplasmy, seeds from these plants were plated on spectinomycin and streptomycin and considered homoplasmic for the selection cassette when all seedlings were indistinguishable from the wild type (WT) grown on non-selective medium (Bock, 2001). To ultimately confirm homoplasmy and stability of the introduced mutations, ∼20 of these seedlings were pooled and sequenced for the desired mutations in *atpB* via PCR amplification and Sanger sequencing. Plant lines with single peaks at the site(s) of the desired mutation(s) were considered homoplasmic (Figure 1A and Supplemental Figure S1). DNA from pooled seedlings was also used for final Southern blot analysis to demonstrate the homoplasmic presence of the selectable marker gene *aadA* in the plastid genomes by a 1.2 kb increase in band size using a probe against the *psaB* gene (Figure 1B). At least three independently transformed transplastomic lines were isolated for each mutation. To assay the effect of the mutations on transcript abundance, we performed a northern blot for the *atpB* transcript with total RNA isolated from young leaves of representative lines for each mutation. This analysis revealed that there is no apparent reduction in transcript abundance (Figure 1C), indicating there are no apparent changes in transcription or transcript stability due to a specific mutation.

### Growth of transplastomic *atpB* mutants

**Figure 2:**
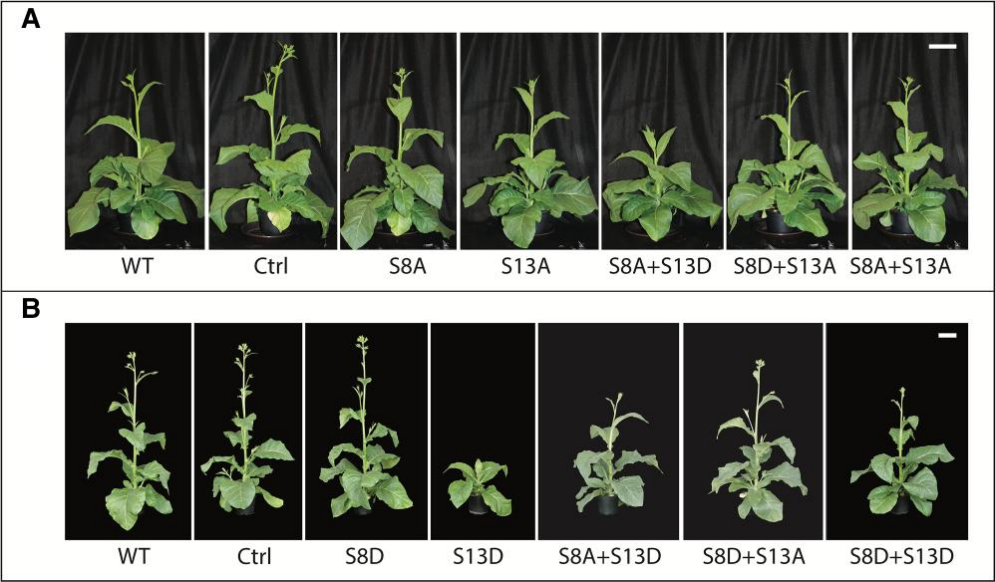
Representative growth of mutants harboring mutations in the N-terminal phosphorylation sites of ATPβ. Serine to alanine (A) or aspartic acid (B) substitutions on either S8 or S13. Scale bars represents 10 cm for each panel. Plants in panels (A) and (B) were grown in separate batches and subsequently photographed on separate days. Background subtraction was applied to images in panel (B). Lines used: (A) *N. tabacum* “Petit Havana” (WT), Ctrl (*aadA* control line), S8A-7A, S13A-18B, S8A + S13D-102A, S8D + S13A-14B, S8A + S13A-8A; (B) WT, Ctrl, S8D-6A, S13D-87A, S8A + S13D-102A, S8D + S13A-168A, S8D + S13D-12B.

As alterations in the activity of ATP synthase should impact the extent of *pmf* leading to changes in feedback regulation of electron transfer (Kanazawa and Kramer, 2002; Rott et al., 2011), we tested the hypothesis that modifications of S8 or S13 of ATPβ would alter plant growth due to altered feedback regulation in the light. Figure 2A shows representative growth of mutants harboring an alanine substitution. It appears that there is no defect in growth with one exception; S8A + S13D leads to a slower growing plant. As the S8A mutant and the S13A mutant have no growth impairment, this is likely due to the aspartic acid substitution for S13. Indeed, in Figure 2B we see representative growth for mutants harboring aspartic acid substitutions, and here we can clearly see that growth is only impaired when the aspartic acid replaces S13, with the single S13D mutation having the largest growth delay.

### Steady state photosynthesis in the *atpB* mutants

**Figure 3:**
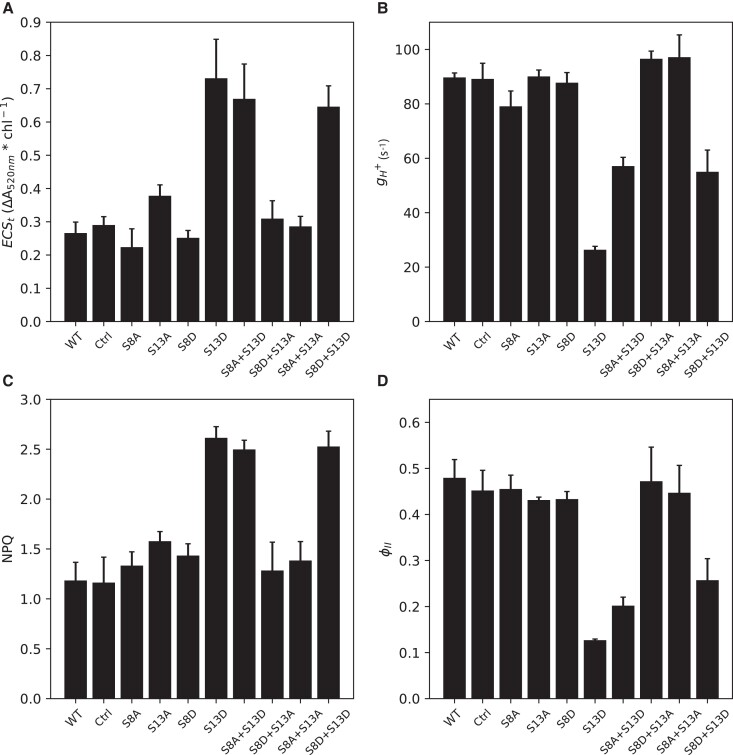
Steady-state photosynthetic parameters of ATPβ mutants in 400 μmoles photons m^−2^ s^−1^. A, Total light to dark amplitude of the electrochromic shift at 520 nm (ECS*_t_*); B, Rate constant of proton efflux (*g*_H_^+^); C, Nonphotochemical chlorophyll fluorescence quenching (NPQ); D, quantum yield of photosystem II (ϕ_II_). Data represents mean +/− SD, *n* = 3. Lines used: *N. tabacum “*Petit Havana” (WT), Ctrl (*aadA* control line), S8A-31A, S13A-7B, S8D-29A, S13D-11A, S8A + S13D-23B, S8D + S13A-91A, S8A + S13A-8A, S8D + S13D-12B.

Because downregulation of photosynthesis due to altered ATP synthase activity was the most likely explanation for impaired growth in the mutants harboring the S13D mutation, we expected that we would see this downregulation reflected in photosynthetic parameters associated with *pmf* and PSII activity. To test this idea, we used chlorophyll *a* fluorescence to probe photosystem II (Baker, 2008), and the electrochromic shift (ECS) of thylakoid membrane embedded pigments in response to membrane potential (Baker et al., 2007) to probe the *pmf*. We hypothesized that the slower growing mutants (i.e. those with the S13D mutation) would have a decreased rate constant for proton efflux (*g*_H_^+^) which is a measure of ATP synthase activity, resulting in increased *pmf*, increased NPQ, and decreased photosynthetic efficiency (ϕ_II_). By taking the total extent of the dark to light changes of the ECS, we can calculate relative steady state *pmf* (ECS*_t_*, Figure 3A), and by fitting this decay to a first order exponential decay, we can calculate *g*_H_^+^ (Figure 3B) (Baker et al., 2007). After steady state illumination with ∼400 μmol photons m^−2^ s^−1^, plants with the S13D mutation showed increased relative total *pmf* (ECS*_t_*) (∼150% increase for all S13D mutants; Figure 3A). This can be attributed to the decrease in *g*_H_^+^ seen for S13D plants (Figure 3B). The largest decrease in *g*_H_^+^ is seen in the single S13D mutant (∼70% decrease), while the decrease in *g*_H_^+^ was comparable between the S8A + S13D and S8D + S13D mutants (∼35% decrease). This decrease in *g*_H_^+^ resulted in an increase in feedback control in the form of NPQ (∼110% increase for all S13D mutants; Figure 3C) and decreased photosynthetic efficiency (∼70%, 60%, and 50% decrease for S13D, S8A + S13D, and S8D + S13D, respectively; Figure 3D), supporting our hypothesis that altered ATP synthase kinetics are the likely cause for the decreased growth of S13D mutants seen in Figure 2.

### ATP synthase protein content in transplastomic *atpB* mutants

**Figure 4:**
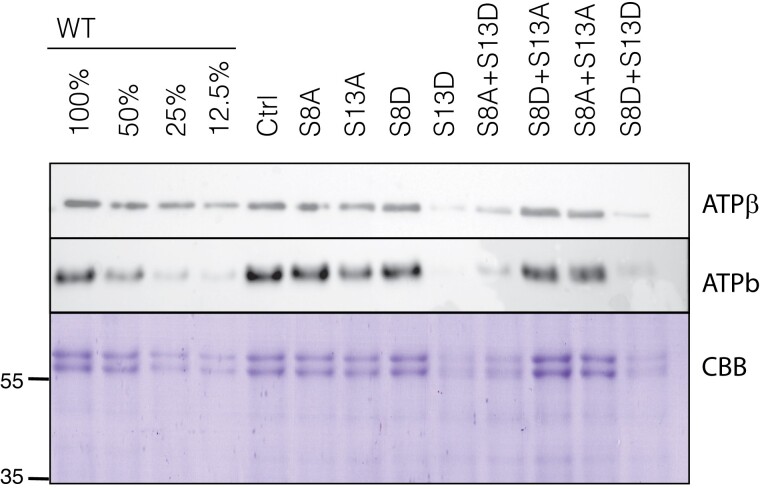
Immunoblot analysis of CF_o_ (ATPb) and CF_1_ (ATPβ) accumulation in isolated thylakoids of ATPβ mutants. ATPβ content is confirmed with Coomassie Brilliant Blue (CBB) staining of the α/β subunits (∼60 kDa). A dilution of 100%–12.5% of WT protein sample was loaded for comparison. Lines used: *N. tabacum* “Petit Havana” (WT), Ctrl (*aadA* control line), S8A-31A, S13A-7B, S8D-29A, S13D-11A, S8A + S13D-23B, S8D + S13A-91A, S8A + S13A-8A, S8D + S13D-12B.

The decreased growth and photosynthetic rates in the mutants harboring the S13D mutation suggested two hypotheses: 1) the phosphorylation state of ATPβ has a regulatory function, as previously suggested (Reiland et al., 2009), changing the specific activity of the complex; or 2) the phosphorylation state alters ATP synthase content, which alters the aggregate or realized activity. A previous study determined at least 50% of ATP synthase content can be lost without a defect in steady state activity, as measured by *g*_H_^+^ (Rott et al., 2011), suggesting that the overall ATP synthase activity is, to some extent, independent of total content, e.g. by regulating pools of active and inactive complexes. However, to rule out protein content changes as a cause for the decreased *g*_H_^+^ in the S13D plants, we quantified ATP synthase content of a CF_1_ subunit (ATPβ) and a CF_o_ subunit (ATPb, encoded by *atpF*) by tricine-SDS PAGE and western blot analyses (Figure 4).

While the alanine mutations led to no apparent change in the amount of ATPβ, this CF_1_ subunit accumulates to a lesser extent in mutants with the S13D substitution. ATPβ content in the S8A + S13D and S8D + S13D mutants is ∼25% of WT and control plants, while the single S13D mutation leads to a reduction in ATPβ content to ∼12.5% of wild type levels. Since the antibody target (ATPβ) is our point of mutation, the western blot results were confirmed by Coomassie blue staining (Figure 4) where the α/β bands of the CF_1_ are seen clearly just above 55 kD. Further, the CF_o_ subunit (ATPb) accumulates similarly, with only the S13D mutations leading to a decrease in content (∼25% of WT for the S8A + S13D and S8D + S13D, and ∼12.5% for S13D). Considering these quantifications, we were therefore unable to rule out that loss of ATP synthase in the S13D mutants is the cause of the altered steady state *g*_H_^+^. It also appears likely that the stronger phenotype in the single mutation of S13D is due to this mutation leading to lower protein accumulation than the S8A + S13D and S8D + S13D mutants.

### ATP synthase dark inactivation

The phosphorylation of ATPβ on residues S8 or S13 has previously been reported at the end of a long dark period (Reiland et al., 2009), which led the authors of that study to propose that phosphorylation is involved in the light to dark regulation of ATP synthase to prevent ATP hydrolysis in the dark. We set out to test this hypothesis by determining the inactivation kinetics of ATP synthase from light to dark. To this end, we measured several parameters involving ATP synthase activity that may change from light to dark. The best-known change in ATP synthase during a light to dark transition is the oxidation of the regulatory thiols on the γ subunit. This oxidation leads to a decrease in the rate constant of the ECS decay after a weak flash in the dark (Kramer et al., 1990; Wu et al., 2007). Similarly, if phosphorylation plays a role in light to dark inactivation of ATP synthase, we might expect a decrease in the rate constant of this decay at earlier time points in the phosphomimic lines, and later time points in the alanine substitution lines. We thus applied a weak actinic flash to light-adapted leaves in increasing time increments after the constant illumination had been turned off and calculated the lifetime (τ_ECS_) of the decay from a first order exponential fit.

ATP synthase is not only redox regulated, but also requires *pmf* for activation (Kramer and Crofts, 1989). In prolonged dark, the threshold of *pmf* for activation (*pmf*_t_) increases as a function of time (Kramer and Crofts, 1989). It is not yet known what factors lead to an increase in *pmf*_t_ in the dark, but phosphorylation has not been ruled out. To test the hypothesis that the phosphorylation state of S8 and S13 on ATPβ increases the *pmf*_t_ in the dark, we gave a multiple turnover actinic flash and monitored the ECS decay kinetics in the leaves of our set of phosphorylation mutants. After the flash, the first derivative of the fast phase has a linear relationship with the amplitude of the flash induced ΔA_520 nm_, and the slope of this relationship is proportional to ATP synthase activity (*g*_H_^+^_d_), and the x-intercept is proportional to *pmf*_t_ (Kramer and Crofts, 1989). We grouped all lines according to substitution (i.e. A or D) and position (i.e. S8 or S13) to see which site/substitution resulted in an altered dark adaptation phenotype.

**Figure 5:**
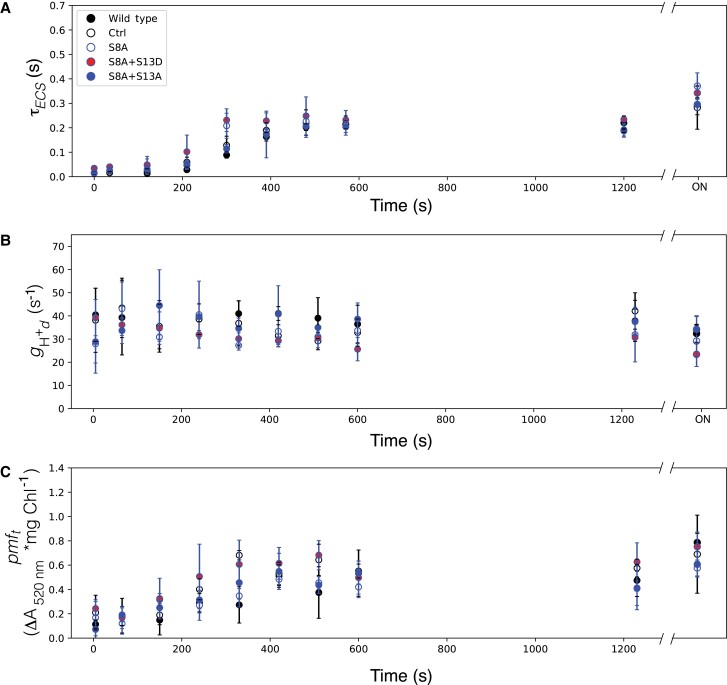
Kinetics of ATPβ -S8A mutants in the dark after illumination. A, Lifetime of ECS decay (τ_ECS_) after a weak actinic flash, reporting the oxidation state of ATPγ. B, transthylakod proton conductivity in the dark (*g*_H_^+^_d_) reflecting the dark adapted activity of ATP synthase; C, threshold of *pmf* activation (*pmf_t_*). Data represents mean +/− SD, *n* = 3. Lines used: *N. tabacum* “Petit Havana” (Wild type), Ctrl (*aadA* control), S8A-31A, S8A + S13D-23B, S8A + S13A-8A.

**Figure 6:**
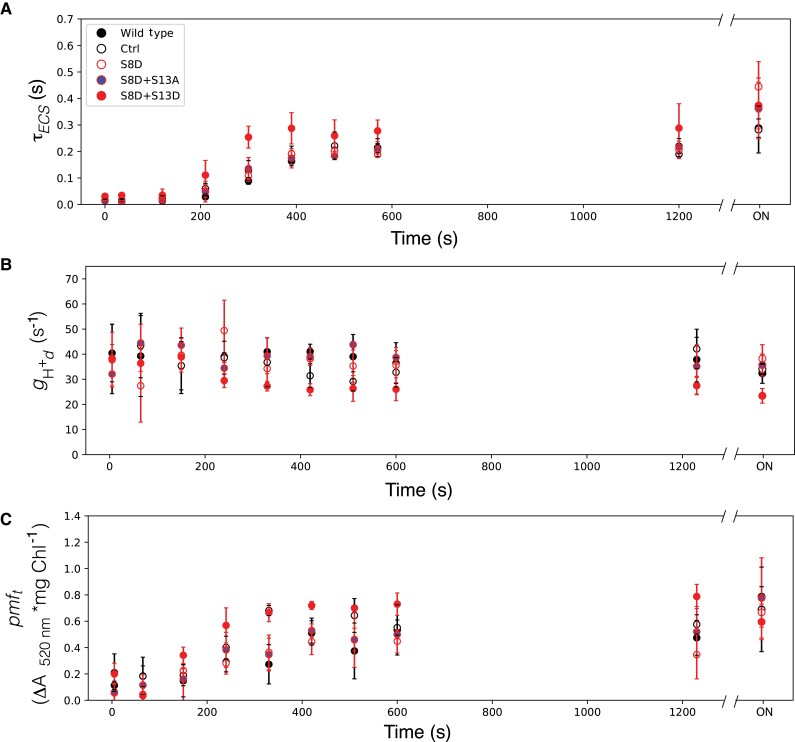
Kinetics of ATPβ-S8D mutants in the dark after illumination. A, Lifetime of ECS decay (τ_ECS_) after a weak actinic flash, reporting the oxidation state of ATPγ. B, transthylakod proton conductivity in the dark (*g*_Hd_^+^) reflecting the dark adapted activity of ATP synthase; C, threshold of *pmf* activation (*pmf_t_*). Data represents mean +/− SD, *n* = 3. Lines used: *N. tabacum* “Petit Havana” (Wild type), Ctrl (*aadA* control), S8D-29A, S8D + S13A-91A, S8D + S13D-12B.

Figures 5 and 6 show dark inactivation parameters for all mutants harboring the S8, A and S8D substitutions, respectively. We see that τ_ECS_ increases as expected, with the wild type and the control reaching ∼2/3 maximum between 8–10 min (Figures 5 and 6, A). This oxidation state is maintained until prolonged darkness, where in the middle of the night (overnight, ON) the τ_ECS_, and thus γ-oxidation reaches maximum. There is no clear agreement in τ_ECS_ deviation from wild type and control kinetics for lines with S8A or S8D. In the wild type and control plants, *g*_H_^+^_d_ (Figures 5 and 6, B) is constant at all time points in the dark (ON). As seen with τ_ECS_, there is no clear deviation from wild type and control kinetics caused by the S8A or S8D substitutions. Finally, *pmf*_t_ (Figures 5 and 6, C) increases with similar kinetics of τ_ECS_ in WT and control plants, with no clear differences in kinetics caused by either S8 substitution.

**Figure 7:**
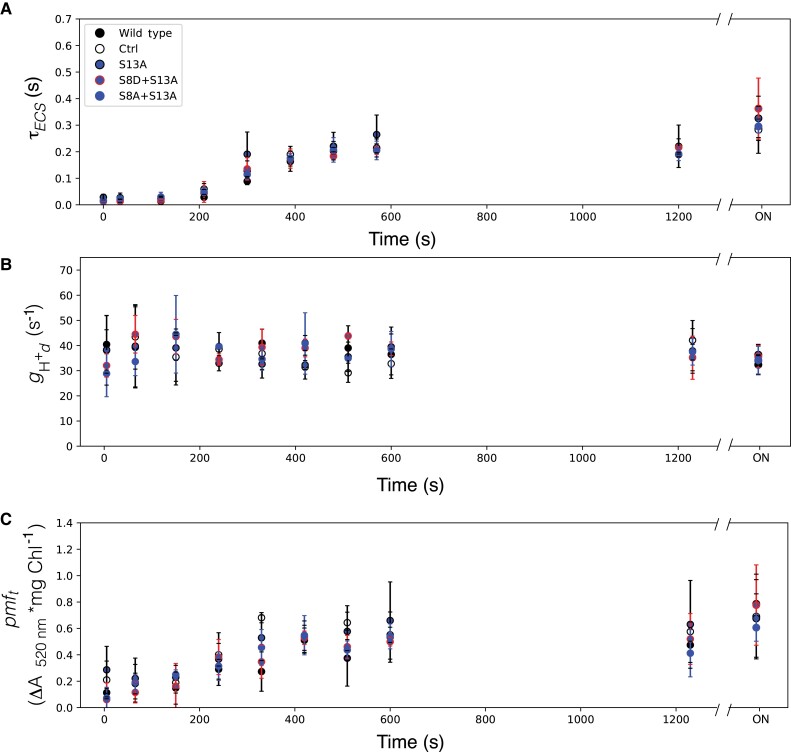
Kinetics of ATPβ-S13A mutants in the dark after illumination. A, Lifetime of ECS decay (τ_ECS_) after a weak actinic flash, reporting the oxidation state of ATPγ. B, transthylakod proton conductivity in the dark (*g*_Hd_^+^) reflecting the dark adapted activity of ATP synthase; C, threshold of *pmf* activation (*pmf_t_*). Data represents mean +/− SD, *n* = 3. Lines used: *N. tabacum* “Petit Havana” (Wild type), Ctrl (*aadA* control), S13A-7B, S8D + S13A-91A, S8A + S13A-8A.

Figure 7 shows dark inactivation parameters for all mutants harboring the S13A substitutions. The S13A substitution results in all plants matching WT and control τ*_ECS_* (Figure 7A), *g*_H_^+^_d_, (Figure 7B), and *pmf*_t_ kinetics (Figure 7C). This suggests that elimination of this phosphorylation site has no impact on the dark inactivation of ATP synthase.

**Figure 8:**
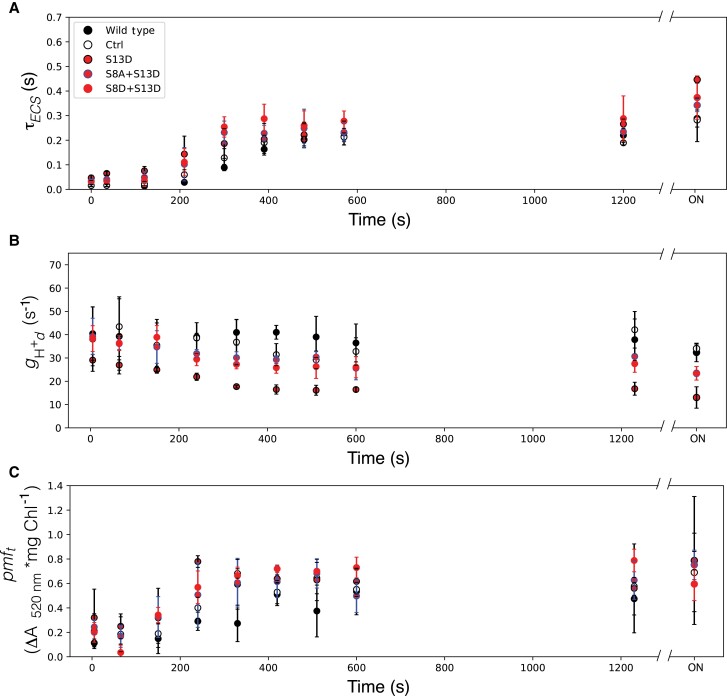
Kinetics of ATPβ-S13D mutants in the dark after illumination. A, Lifetime of ECS decay (τ_ECS_) after a weak actinic flash, reporting the oxidation state of ATPγ. B, transthylakod proton conductivity in the dark (*g*_Hd_^+^) reflecting the dark adapted activity of ATP synthase; C, threshold of *pmf* activation (*pmf_t_*). Data represents mean +/− SD, *n* = 3. Lines used: *N. tabacum* “Petit Havana” (Wild type), Ctrl (*aadA* control), S13D-11A, S8A + S13D-23B, S8D + S13D-12B.

However, we see that when the S13D mutation is present, the prolonged intermediate oxidation state of ATPγ was reached within 5 min of the switch from light to dark, compared to the ∼10 min in the wild type and control plants. This finding indicates that γ-oxidation is accelerated in the S13D plants (Figure 8A). There seems to be no clear trend to the final extent of γ-oxidation (τ_ECS_ at time = ON) for either mutation or site, thus the variation was likely not reflective of anything related to the mutations or protein content. ATP synthase dark activity, *g*_H_^+^_d_, is lower in the S13D mutants than in the wild type and the *aadA* control lines (Figure 8B), with the strongest decrease from wild type activity seen in the S13D single mutant. While in the WT, maximum *pmf*_t_ was reached after ∼400 s in the dark and did not appear to increase to any substantial extent until the ON timepoint, plants harboring the S13D mutation appear to have a faster increase in *pmf*_t,_ reaching maximum *pmf*_t_ after around 5 min (Figure 8C). However, the ON differences between the wild type and control plants compared to the S13D mutants were unremarkable.

**Figure 9:**
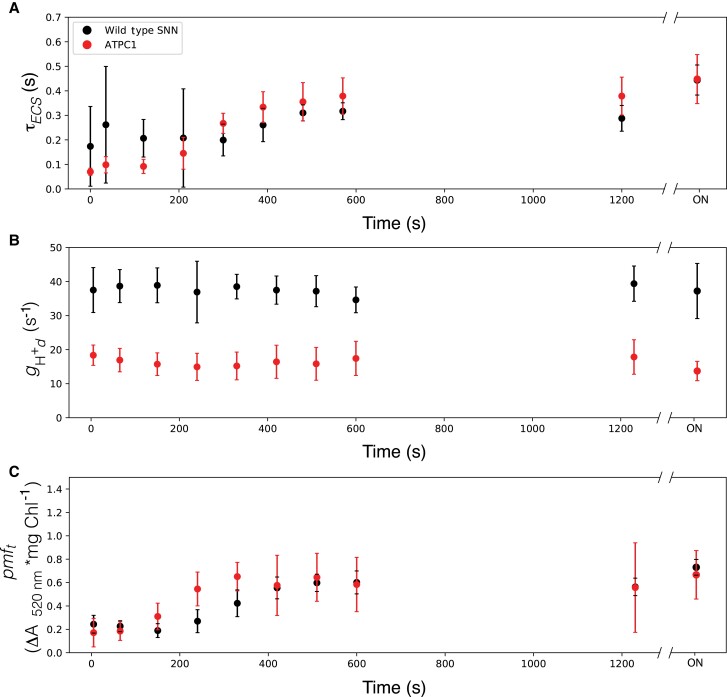
Kinetics of *ATPC1* knock-down mutants in the dark after illumination. A, Lifetime of ECS decay (τ_ECS_) after a weak actinic flash, reporting the oxidation state of ATPγ. B, transthylakod proton conductivity in the dark (*g*_Hd_^+^) reflecting the dark adapted activity of ATP synthase; C, threshold of *pmf* activation (*pmf_t_*). Data represents mean +/− SD, *n* = 3. Lines used: *N. tabacum “*Samsun-NN” (Wild type SNN) and *ATPC1* antisense mutant (ATPC1).

Since the only defect in ATP synthase inactivation kinetics was seen in plants with decreased ATP synthase content, we hypothesized that the altered kinetics were due to a decrease in protein content, and not enzyme-level regulation. We tested this hypothesis by performing similar experiments with the *ATPC1* antisense plants described in (Rott et al., 2011). These plants accumulate ∼12% of ATP synthase, comparable in protein content with our most strongly defective S13 phosphomimic mutation. In these *ATPC1* antisense plants, we observed a faster increase in τ_ECS_ (Figure 9A) in the dark, indicating that the decrease in ATP synthase content might explain the increased rate of ATPγ oxidation in our S13 phosphomimic lines. Additionally, a decrease in *g*_H_^+^_d_ was seen in the *ATPC1* knock-downs (Figure 9B), qualitatively mimicking the phenotype seen in the single S13D ATPβ mutant (Figure 8B). The *ATPC1* antisense lines also showed an earlier increase in *pmf*_t_ in the dark (Figure 9C). These data, taken together with the lack of a detectable phenotype of the S13A plants (Figure 7), suggest that the phenotypes seen for the S13D plants in the dark were not a result of the mimicked phosphorylation state, but instead, were caused by decreased ATP synthase content that results from the S13D mutation.

### ATP synthase content in response to drought

**Figure 10:**
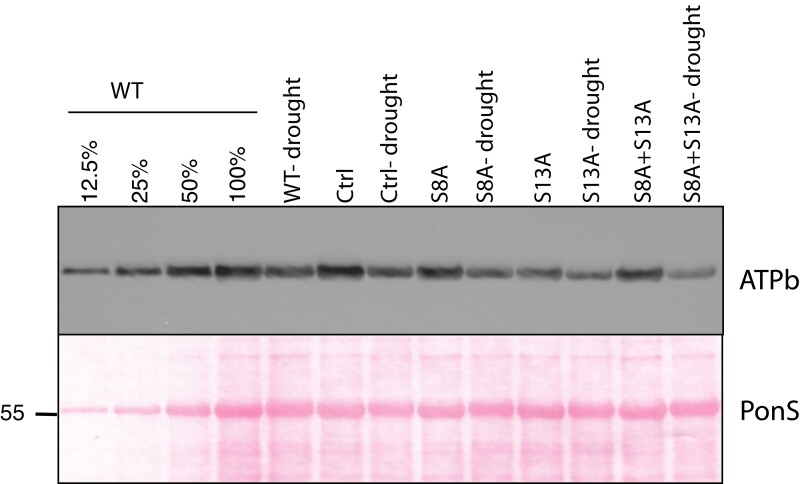
Immunoblot analysis of CF_o_ (ATPb) protein accumulation in total leaf protein samples from well-watered and drought stressed plants. A 100%–12.5% dilution of non-stressed WT protein sample was loaded for comparison. Lines used: *N. tabacum* “Petit Havana” (WT), Ctrl (*aadA* control line), S8A-12A, S13A-29A, S8A + S13A-14B. PonS: Ponceau S staining of the membrane after blotting.

In the absence of any substantial change to ATP synthase dark inactivation in our non-phosphorylatable mutants, we considered conditions in which ATP synthase activity or content is expected to be altered to test the hypothesis that phosphorylation is involved in modulating activity under stress conditions. Known conditions under which ATP synthase activity is altered include response to drought (Kohzuma et al., 2009), low CO_2_ (Kanazawa and Kramer, 2002), and phosphate limitation (Takizawa et al., 2008). Under drought stress, ATP synthase content and activity is decreased (Kohzuma et al., 2009), and ATPβ has been shown to be phosphorylated by CK-II in vitro (Kanekatsu et al., 1998), a protein kinase proposed to be involved in drought stress responses (Vilela et al., 2015). These observations led us to hypothesize that the phosphorylation of ATPβ could be a signal for the decrease in ATP synthase content in response to drought. To test this idea, we drought-stressed our mutants harboring the S to A mutations and measured ATPβ content by western blot analysis. Figure 10 shows that all mutants showed decreases in ATPβ content after drought stress, regardless of the mutation, indicating that phosphorylation of the S8 or S13 residues is unlikely to be the only signal for decreased ATP synthase content in response to drought.

### Kinetics of ATPb turnover in *atpB* mutants

**Figure 11:**
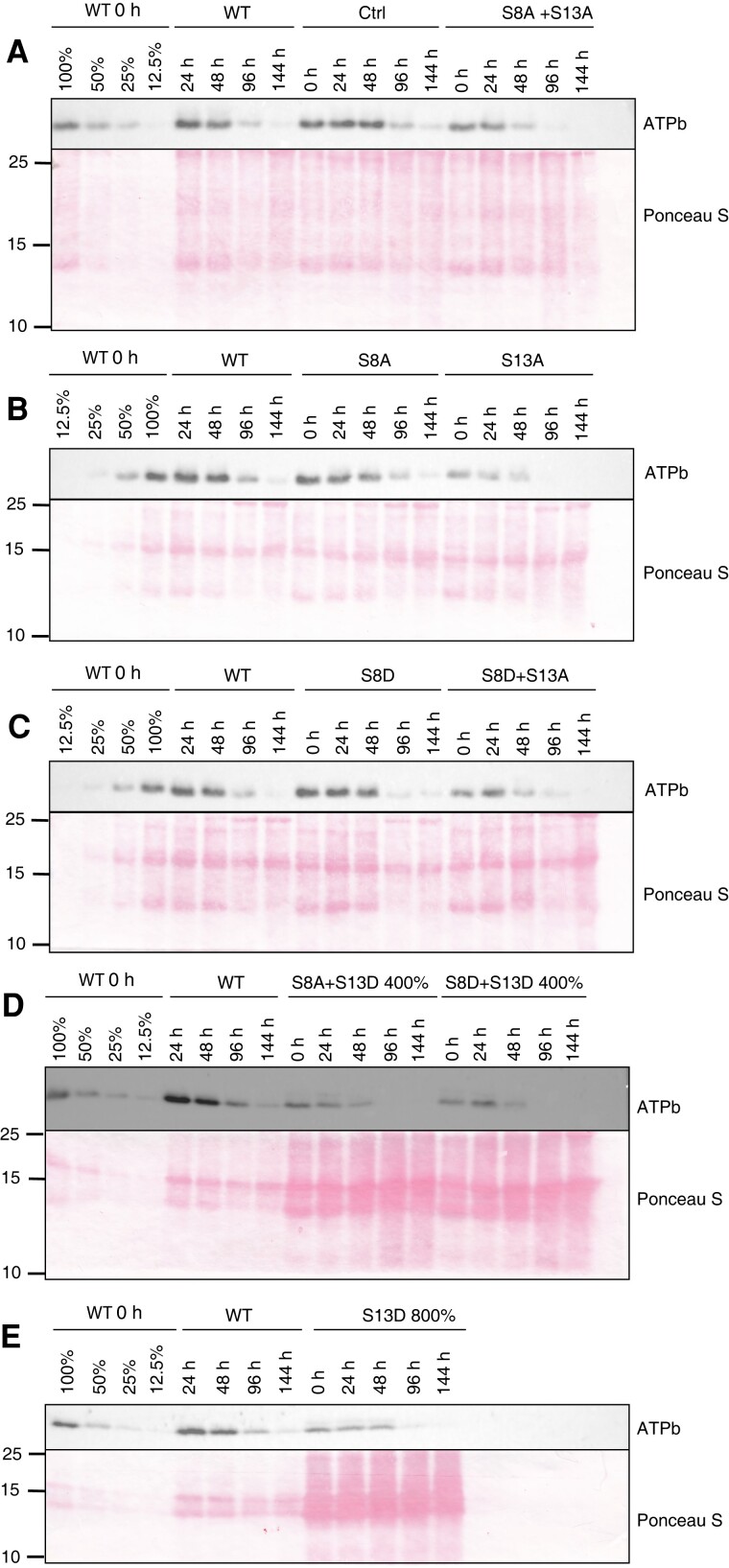
Time course of ATPb content after infiltration of leaf discs with 3 mM lincomycin. A 100%–12.5% dilution of WT 0 hour (h) protein sample was loaded for comparison. A–C, Representative mutant lines accumulating WT levels of ATP synthase. D and E, Representative mutant lines accumulating substantially less ATP synthase than the WT. Lines used: *N. tabacum* “Petit Havana” (WT), Ctrl (*aadA* control line), S8A-7A, S13A-29A, S8D-16B, S8D + S13A-91A, S8A + S13A-14B, S13D-11A, S8A + S13D-102A, S8D + S13D-8B.

To understand the decreased accumulation of ATP synthase subunits in our S13 mutants, we generated two hypotheses. 1) The S13D substitution leads to an increase in the degradation rates of ATP synthase, and 2) the S13D substitution leads to a decrease in the rate of ATP synthase synthesis. To test for altered turnover rate of ATP synthase, we infiltrated leaf discs with lincomycin and sampled at 24, 48, 96, and 144 h after infiltration. Total protein samples from our time course were then blotted and hybridized to an antibody against ATPb (encoded by *atpF*) (Figure 11) as a marker for ATP synthase content. We hypothesized that we would see a higher rate of ATPb degradation in the S13D mutants and a slower rate of ATPb degradation in the S13A mutants, if phosphorylation was signaling for protein turnover. Figure 11 shows the ATPb degradation kinetics for our ATBβ mutants that show wild type ATP synthase accumulation. Degradation kinetics appear to be wild type-like for the control plants and the S8A + S13A mutant (Figure 11A), the S8A and S13A mutants (Figure 11B), and the S8D and S8D + S13A mutants (Figure 11C). To see the degradation kinetics of ATPb in our low accumulating *atpB* mutants (i.e. mutants harboring the S13D mutation), we increased the protein loading to levels that would give us near wild type signals for ATPb (400% of WT for S8A + S13D and S8D + S13D, and 800% of WT for S13D; Figure 11, D and E, respectively). On a total protein basis, ATPb did not appear to decrease at a substantially different rate for the S13D mutants. We, therefore, rejected the hypothesis that changes in ATP synthase content in our phosphomimic lines is due to increased protein degradation rates. Instead, it seems more likely that the lower ATP synthase content in the S13D mutants was caused by defects in synthesis and/or assembly of the complex, consistent with the observed lack of defects in mRNA expression or stability (Figure 1C).

### Complex accumulation in *atpB* mutants

The dearth of changes in transcript accumulation and degradation rate in the *atpB* mutants prompted us to analyze the assembly of the ATP synthase by BN-PAGE in thylakoid samples from the wild type, the control plants and the *atpB* mutants S13D, S8A + S13D and S8D + S13D. One-dimensional gels revealed similar complex distribution for photosystem II, photosystem I, the cytochrome *b*_6_*f* complex and the LHCII monomer and trimers. Despite similar content of ATP synthase in control plants and the wild type, we noted lower accumulation of fully assembly ATP synthase in all the S13D *atpB* mutants (Supplemental Figure S2A). To confirm these observations, we evaluated the accumulation of ATPβ by immunoblot analyses in one-dimensional gels (Supplemental Figure S2B). In wild type and control plants, the main signal obtained for ATPβ corresponded to the fully assembled ATP synthase. While all the mutants exhibited decreased assembled ATP synthase, wild type and control plants exhibited high accumulation of ATPβ in intermediate complexes, with a prominent band below the monomeric LHCII (mLHCII) not observed in the *atpB* mutants (Supplemental Figure S2B). Additionally, all the genotypes exhibited a high molecular ATPβ -containing complex at the top of the fully assembled ATP synthase (complex *). To better characterize the distribution of the complex intermediates, we employed reducing second dimensional polyacrylamide gels for the control plants and the *atpB* mutants (Supplemental Figure S2C). Similar to our one-dimensional immunoblots, all genotypes exhibited the high molecular weight complex that contained ATPβ (complex *), and decreased accumulation for the fully assembled ATP synthase in the *atpB* mutants. In the control plants, we identified at least four different complex intermediates containing ATPβ (assembly intermediates 1–4), in agreement with the observations reported by (Rühle et al., 2014). By contrast, all the *atpB* mutants exhibited decreased accumulation for all the intermediates, with intermediate 4 being almost undetectable. Overall, these results suggest that the phosphomimic at S13 affects the accumulation of early assembly intermediates of the ATP synthase.

### Photosynthesis under dynamic light conditions in the *atpB* mutants

As a central regulator of the thylakoid proton circuit, ATP synthase has a critical role in feedback regulation of light harvesting and electron transfer under dynamic conditions. To assess if our mutations led to defects in light acclimation, we subjected our plant lines to three types of light conditions over five days using a fluorescence imaging platform described in (Cruz et al., 2016). The Dynamic Environmental Photosynthetic Imaging (DEPI) platform allowed us to perform relatively high-throughput chlorophyll *a* fluorescence phenotyping of our *atpB* mutants to identify possible impacts on feedback regulation imposed by our mutations. Plants from one representative line of each mutation were imaged over the course of five days. The light conditions of the entire time course are shown in panel A of Supplemental Figures S3–S6. In the wild type and the control plants, as well as all of the *atpB* mutants, total NPQ and ϕ_II_ are relatively steady through the course of the flat days (days 1 and 4, Supplemental Figures S3 and S4, B–J). While all mutants harboring the S13D mutation had some increase in NPQ during the flat days, the most substantial increase was seen in the single S13D mutant (accumulating 12.5% of WT levels of ATP synthase, Figure 4). The S13D single mutant had a ∼ 500% increase in NPQ, as well as a ∼40% decrease in ϕ_II_. NPQ also increased during the flat days in the *ATPC1* antisense plants, with similar ATP synthase accumulation levels [(Rott et al., 2011), Supplemental Figure S6B], suggesting the increase in photoprotection in the S13D mutants are due to decreased content of ATP synthase. Since the primary component of NPQ that would be impacted by altered ATP synthase is the pH-dependent NPQ, *q*_E_, we also integrated short (2-min) dark intervals during our experiment to allow a relative measure of *q*_E_ (Supplemental Figures S5 and S6D). When looking specifically at *q*_E_, we see an increase in *q*_E_ for all mutants harboring the S13D mutation, with the strongest in the S13D single mutant. In the *ATPC1* antisense plants, we see that the flat days have similar increases of *q*_E_ over the wild type, suggesting ATP synthase content is a large contributing factor.

During the sinusoidal day (day 2), all plants show the expected increase in NPQ and decrease in ϕ_II_ with increasing light intensity, as well as the reciprocal relaxation as light intensity decreases (Supplemental Figures S3 and S4, B–J). In all plants harboring the S13D mutation, these curves are broader, indicating a stronger NPQ response to light, resulting in decreased ϕ_II_ (Supplemental Figures S3 and S4, D–G). Similar results are seen in the *ATPC1* antisense plants (Supplemental Figure S6, B and C), suggesting that ATP synthase content of the S13D mutants is a contributing factor to the NPQ response during the sinusoidal day. Further support of this conclusion comes from the observation that the S8A + S13D and S8D + S13D plants (containing ∼25% ATP synthase, Figure 4) had slightly later NPQ and ϕ_II_ response curves when compared to the single S13D mutant during the sinusoidal day. While in most of the plant lines, *q*_E_ mimicked the NPQ response curve, in the plants harboring the S13D mutation, and in the *ATPC1* antisense plants, *q*_E_ first increased and then decreased during the high light portion of the day (Supplemental Figures S5 and S6D), suggesting slowed relaxation kinetics of *q*_E_, or accumulation of a secondary quencher such as photoinhibition (*q*_I_), which has been shown to accumulate in response to high *pmf* (Davis et al., 2016). Finally, fluctuating light conditions were used to draw out recalcitrant photosynthetic phenotypes, and for this purpose, we exposed our plants to multiple days of fluctuating light (day 3 and 5). Under these conditions, the observed photosynthetic phenotypes were similar to those of the sinusoidal day (Supplemental Figures S3–S5, B–J, and S6), indicating the phenotypes observed are due to the intensity of the light exposure, and not fluctuations.

While there were subtle differences in photosynthetic parameters in ATPβ mutants other than those harboring the S13D mutation, the light responses during the sinusoidal day followed those typical for the wild type and the control line, and did not have a clear pattern indicating an impact from any specific mutation.

## Discussion

To assess the role of N-terminal phosphorylation of ATPβ in photosynthetic regulation, we used a set of mutants locked in specific phosphorylation states. We saw defects in ATP synthase in the light and altered inactivation kinetics in the dark associated with decreased protein accumulation in all mutants harboring the S13D mutation. When we tested the hypothesis that the phosphorylation state of ATPβ serines 8 and 13 contributed to the dark inactivation of ATP synthase as proposed in (Reiland et al., 2009), we only saw changes in S13D plants (Figure 8). These plants had faster ATPγ oxidation and appearance of increased *pmf*_t_, and in the case of the single S13D mutation, lower ATP synthase activity in the dark (*g*_Hd_^+^). We demonstrated that this is due to loss of ATP synthase complex, and not dynamic regulation of the complex, by comparing the inactivation kinetics of an ATP synthase knock-down mutant (Figure 9, (Rott et al., 2011)), which yielded qualitatively similar results. Therefore, we rejected the hypothesis that phosphorylation contributes to inactivation in the dark. Instead, the main light/dark switch in the chloroplast ATP synthase is likely oxidation of ATPγ, as has been demonstrated elsewhere (Kohzuma et al., 2012). In fact, it seems that the ATPγ oxidation state is likely the regulator of the *pmf* threshold of activation (*pmf*_t_) for ATP synthase, as both of these are altered on the same timescale when ATP synthase content is decreased. In general, the amount of ATP synthase in the chloroplast should not alter the E_m_ of ATPγ. Instead, the faster oxidation of ATPγ could be explained by a decrease in electron transfer that might be expected from the decrease in ϕ_II_ (Figure 3) we see in the S13D plants. A decrease in the bulk flow of electrons may lower the electron buffering capacity of the stroma in the dark, leading to quicker oxidation of ATPγ and likely other redox-regulated proteins in the chloroplast.

Due to the decrease in ATP synthase content in our ATPβ S13D mutants (Figure 4), we hypothesized that phosphorylation was a signal for protein degradation in the dark. However, in the presence of lincomycin, there was no substantial differences in the apparent degradation rate of the CF_o_ subunit ATPb in any of our mutants (Figure 11). In addition, our mutants accumulate wild type-like transcript levels (Figure 1C), suggesting that the defect in protein accumulation is either due to reduced translation or impaired assembly. It seems possible that degradation involves a chloroplast-encoded factor that would be inhibited by lincomycin, such as the P1 subunit of the Clp protease (Moreno et al., 2018), but ATP synthase is currently not thought to be a target of Clp. Therefore, with the information we have on hand, we must reject our hypothesis that the phosphorylation state of ATPβ-S13 is a signal for degradation.

As the rate of translation is unlikely to be altered due to a change in a single internal codon, the remaining point for a defect in complex accumulation is assembly. Our BN-PAGE and two-dimensional gels show lower accumulation of the assembly intermediate 4 in the S13D, S8A + S13D and S8D + S13D mutants compared to wild type and control plants, suggesting that the phosphorylation of ATPβ at the N-terminal position S13 may hinder the early assembly of the ATP synthase. In view of the lower accumulation of the assembly intermediate 4 in the *atpB* mutants, we speculate that the phosphorylation of S13 could interfere with the association of chaperones or assembly factors at an early stage of ATP synthase biogenesis. Therefore, the phosphorylation of S13 could be one of the mechanisms to regulate the content of the ATP synthase in the dark. CF_o_ and CF_1_ subunits appear to be lost stoichiometrically (Figure 4). There is no evidence suggesting misassembly of ATP synthase in the S13D mutants, in that misassembly would likely lead to a leaky enzyme and result in faster ATPγ oxidation kinetics in Figure 8A (as discussed in (Strand et al., 2017)). Thus, the defect to accumulation appears to be only in the accumulation kinetics. As of writing, no X-ray crystal or cryo-EM structure has been resolved containing the S8 or S13 residues of CF_1_-β, suggesting that these amino acids lie within a disordered region of the CF_1_. Given this lack of structural data, it is difficult to further hypothesize what, if any, defect the phosphomimics are causing in ATP synthase assembly.

In this manuscript, we ruled out multiple hypotheses for the role of ATPβ N-terminal phosphorylation in ATP synthase regulation, specifically the hypothesis of light-dark regulation and protein turnover. There are other possible roles for this phosphorylation in ATP synthase regulation that have yet to be tested. As ATP synthase is proposed to have multiple regulatory phosphorylation sites, identification of phosphorylation of specific ATP synthase residues under conditions in which activity is known to be altered (e.g. low CO_2_ or P_i_) may be a more productive first approach. It has been proposed that CK-II is a kinase for ATPβ (Kanekatsu et al., 1998; Reiland et al., 2009), and phosphorylation still may serve some yet unknown function in the dark. ATPβ forms part of the catalytic domain mediating ATP synthesis and hydrolysis. If we hypothesize that phosphorylation of these residues is functional and not artifactual or promiscuous, then the question becomes “what occurs at night that would require regulation of the catalytic CF_1_ domain?”. It has been proposed that ATP synthase needs to be inactivated so that dark ATP hydrolysis does not occur (Kohzuma et al., 2012). However, since ATP hydrolysis in the dark is linked to *pmf* formation, and we do not see any *pmf* linked changes in activity that cannot be attributed to protein content, the activity that is being regulated would likely need to be independent of *pmf*. One possible function for phosphorylation in the dark could be to shift the K_M_ of substrate to allow for changes in the activation kinetics of ATP synthase in the morning, however there is no clear evidence for this from our DEPI experiments (Supplemental Figures S3–S5).

An interesting aspect of ATP synthase is that it is regulated primarily post-translationally, and the rate constant of proton efflux has been shown to be unresponsive to decreases in ATP synthase content as low as 50%, suggesting that there is a pool of inactive ATP synthase in the thylakoid (Rott et al., 2011). In that report, the relationship between activity and content remained unknown between 12.5% and 50%. Therefore, the extent of the inactive fraction of the ATP synthase pool was assumed to be 50% due to the linear relationship of activity and protein content between 50% and ∼12.5%. Here we contribute another point to the curve, 25% ATP synthase content, shown for our *atpB* mutants S8A + S13D and S8D + S13D (Figure 4). We see that a 75% decrease in ATP synthase content leads to a ∼40% decrease in activity, while the 87.5% decrease in content in our S13D mutant leads to a ∼75%–80% decrease in activity (Figure 4), similar to the decrease in activity seen in (Rott et al., 2011) with similar ATP synthase content. These data suggest no regulatory role of N-terminal ATPβ phosphorylation in steady-state photophosphorylation, and the simplest explanation for decreased *g*_H_^+^ is the decrease in ATP synthase content. Since we show that ATP synthase activity decreases at 25% content, we suggest an inactive fraction of between 50% and 75%. All else being equal, if we assume that the inactive fraction is 50%, there is a roughly linear relationship between rate constant and protein content when the inactive pool is eliminated. However, if the inactive pool is >50%, the relationship appears more exponential like, which would suggest that the remaining ATP synthase in these mutants are less active than their wild type counterparts, an unlikely scenario, therefore, our data are in clear agreement with the findings of Rott et al. (2011).

In summary, our work reported here suggests a function of amino acid 13 in CF_1_ assembly rather than in the regulation of ATP synthase activity.

## Materials and methods

### Plant material and growth conditions

All experiments were performed in the *Nicotiana tabacum* “Petit Havana” cultivar. Spectroscopic experiments measuring ATP synthase dark inactivation kinetics and high-throughput chlorophyll *a* fluorescence imaging additionally included the *N. tabacum* “Samsun-NN” (SNN) cultivar.

Leaf tissue for DNA analyses came from tobacco plants grown on 0.5 MS medium (6.8 g/L phytoagar, 3% (w/v) sucrose) at 50 μmol photons m^−2^ s^−1^. For RNA and protein analyses, plants were grown in 200 μmol photons m^−2^ s^−1^ with a 16:8 light:dark photoperiod. RNA was extracted from the youngest leaf of plants at 3 weeks after sowing. Spectroscopic analysis and protein extraction was performed on fully expanded leaves (17–20 cm in length) at ∼5 weeks after sowing. For growth comparison, seeds were germinated in 200 μmol photons m^−2^ s^−1^ with a 16:8 light:dark photoperiod for approximately 3 weeks, then transferred to larger pots and grown at 350 μmol photons m^−2^ s^−1^ for approximately 8 weeks. Photos were taken when control plants (transformed with the construct containing the *aadA* marker but no *atpB* mutation) and wild type plants began producing flower buds. Due to space constraints, representative lines harboring the S-to-A mutations at either site and representative lines harboring the S-to-D mutations at either site, were grown in independent experiments.

### Construction of plastid transformation vectors and plastid transformation

A 2018bp region of the tobacco plastid DNA containing the coding sequence for the N-termini of ATPβ and RbcL was amplified by PCR and inserted into cloning vector pMCS5 (MoBiTec) between the PmeI and PacI restriction sites (NEB) using Gibson assembly (Gibson, 2011) and primer pair P1 and P2 (see Supplemental Table S1). The resulting plasmid (pDDS026) was then used as template to introduce all mutations except S8D + S13A by site directed mutagenesis using mismatched primer pairs (Supplemental Table S1). After amplification, the PCR products were digested with the restriction enzyme DpnI (NEB) to eliminate the template plasmid. To obtain the S8D + S13A mutation, a Gibson assembly protocol was employed using primer pairs P1/P17, and P2/P18, with P17 and P18 incorporating the two desired point mutations (Supplemental Table S1). The resulting fragments were inserted into pMCS5 as described for pDDS026. In total, 8 combinations of mutations were generated (Supplemental Table S1) and transformed into *E. coli* strain TOP10. Ampicillin-resistant colonies were sequenced for the desired mutation(s) before being combined with plastid transformation vector pDK308 (Nt-EctWT, (Agrawal et al., 2020)), containing a chimeric *aadA* gene (Svab and Maliga, 1993) as selectable marker gene for chloroplast transformation, and used in biolistic co-transformation experiments as described in (Fuentes et al., 2012; Krech et al., 2013).

### Selection of transplastomic tobacco lines

After particle bombardment, primary transplastomic lines were selected as described in (Caroca et al., 2021). Tobacco plants that appeared heteroplasmic by PCR and Sanger sequencing underwent additional regeneration rounds until they appeared homoplasmic by sequencing. Putative homoplasmic plants were further analyzed by Southern blot for homoplasmic integration of the *aadA* cassette, and lines with a single band shift of 1.2 kb were transferred to soil for seed production. Homoplasmy was confirmed for integration of the *aadA* cassette by plating the resulting seeds on selection medium and Southern blot analysis of DNA from ∼20 pooled seedlings. Lines homoplasmic for the *aadA* displayed a homogeneous population of spectinomycin-resistant progeny (Bock, 2001) and a single band at 5.7 kb in the Southern blot analysis. Homoplasmy for the point mutations was confirmed by PCR amplification and Sanger sequencing of the same DNA as used for Southern blot analysis. Lines were considered homoplasmic when they had a single peak in the sequence chromatograms.

### Isolation of nucleic acids and gel blot analyses

Leaf tissue for DNA and RNA analyses was frozen in liquid nitrogen and pulverized using a steel ball Retsch mill. DNA was extracted according to (Doyle and Doyle, 1987) for Sanger sequencing and Southern blotting. For Southern blot analyses, total genomic DNA was digested with BglII, separated by gel electrophoresis in 0.7% (w/v) agarose gels and blotted onto Hybond N nylon membranes (GE Healthcare). A ∼500 bp probe was produced by PCR amplification (primer pair P19/P20) of part of the *psaB* gene, followed by [α-^32^P]dCTP-labeling by random priming (Multiprime DNA labeling kit; GE Healthcare).

Total RNA extraction and northern blot analysis was performed as described in (Rott et al., 2011) using primer pair 21/22 (Supplemental Table S1) to generate a DNA probe against *atpB*.

### Thylakoid extraction

Crude chloroplasts were extracted from mature leaves (∼15–20 cm in length) as described in (Strand et al., 2016) with the addition of 10 mM sodium ascorbate to all buffers. Chloroplasts were then subjected to an osmotic shock (10 mM HEPES, 5 mM MgCl_2_, 2.5 mM EDTA, 10 mM sodium ascorbate, pH 7.6) on ice for 10 min and thylakoids were pelleted at 3000*×g* for 10 min at 4°C. The pellet was resuspended in the osmotic shock buffer and used for subsequent experiments.

### Protein sample preparation and tricine-SDS PAGE

For western blots based on chlorophyll concentration, thylakoids of the desired chlorophyll concentration were centrifuged at 3000*×g* for 10 min at 4°C and resuspended in a modified Laemmli sample buffer (100 mM Tris-HCl, 4% (w/v) SDS, 8 M urea, 12% (v/v) β-mercaptoethanol, and 20% (v/v) glycerol). For blots based on total protein, leaf punches from mature leaves were ground in liquid nitrogen in a Retsch mill. Protein was then extracted in buffer (100 mM Tricine-KOH, 2 mM MgCl_2_, 10 mM NaCl, 1 mM EDTA, 0.2% Trition-X 100, pH 7.5) and assayed for total protein content (Protein Assay Kit I, Bio-Rad). The protein was then precipitated in 80% (v/v) acetone at −20°C overnight, and further treated identically to the samples loaded based on chlorophyll concentration.

Protein samples were gel electrophoretically separated as described in (Schägger, 2006) through a 4% (w/v) stacking and 10% (w/v) resolving gel at 16°C. Proteins were transferred to a nitrocellulose membrane with 350 mA for 4 h, with the voltage not exceeding 20 V, using a semidry transfer apparatus and transfer buffer containing 48 mM Tris, 39 mM glycine, and 20% (v/v) methanol. Membranes were stained with Ponceau S prior to blocking. Membranes were then rinsed in deionized water and incubated for 1 h in TBST containing 5% (w/v) dry milk as a blocking agent. Antibodies (anti-AtpB for ATPβ; anti-AtpF for ATPb; Agrisera) used to probe the membranes were prepared according the manufacturer’s dilution recommendation, and incubated on the membrane overnight at 4°C. Chemiluminescense from ECL Prime Western Blotting Detection Reagents (GE Healthcare) was detected on the G:Box Chemi XT4 (Syngene).

### BN-PAGE and two-dimensional gel electrophoresis

BN-PAGE was conducted according to (Järvi et al., 2011) with the modifications described in (Sandoval-Ibáñez et al., 2022). Thylakoid membranes equal to 20 μg of chlorophyll were resuspended to a final concentration of 1 μg mL^−1^ chlorophyll in 25BTH20G buffer (25 mM BisTris/HCl (pH 7.0), 20% (w/v) glycerol and 2× cOmplete EDTA free protease inhibitor (Roche)). The samples were then solubilized by adding an equal volume of 2% (w/v) n-dodecyl-β-maltoside (β-DDM) and incubated for 2 min at 4°C in the dark. The samples were centrifuge at 15,000 g for 20 min and the solubilized fraction was mixed with 1/10 volume of loading dye (100 mM BisTris/HCl pH 7.0, 0.5 M epsilon-aminocaproic acid (ACA), 30% (w/v) sucrose and 50 mg mL^−1^ Serva Blue G). Thylakoidal protein complexes were resolved in a 8%–13.9% (w/v) gradient gel. For direct immunodetection in one-dimensional BN-PAGE, the proteins were solubilized with Laemmli buffer (138 mM Tris-HCL pH 6.8, 6 M urea, 22.2% (v/v) glycerol, 4.3% (w/v) SDS and 100 mM DTT) for 1.5 h and blotted onto a PVDF membrane. For the second dimension, the gel strips were treated with Laemmli buffer and resolved in a 12.5% (w/v) SDS-polyacrylamide gel. Immunodetection with the anti-AtpB antibody was conducted as described above.

### In vivo absorption and fluorescence spectroscopy

Measurements were performed on a home-built spectrophotometer described in (Hall et al., 2013). Steady state chlorophyll a fluorescence and absorbance measurements were performed as described in (Strand et al., 2017). Plants were dark adapted for 30 min prior to experimentation. Actinic illumination was 400 μmol photons m^−2^ s^−1^.

To measure ATP synthase inactivation kinetics, two flash-induced relaxation kinetics (FIRK) of the electrochromic shift at 520 nm were performed as described in (Kramer and Crofts, 1989). Tobacco plants were pre-illuminated with 400 μmol photons m^−2^ s^−1^ prior to experimentation. After 15 min of illumination, the light was turned off. First, a weak flash was given at 0, 35, 120, 210, 300, 390, 480, 570, and 1200 s post illumination, and the absorbance change from a green LED filtered with a 520 nm bandpass filter was monitored. The decay of the flash-induced absorbance shift was fit to a first order exponential decay to determine the lifetime (τ*_ECS_*). Second, a sub-saturating flash was given at 5, 65, 150, 240, 330, 420, 510, 500, and 1230 s post illumination, and the absorbance change at 520 nm was monitored. The first derivative of the decay was plotted against the amplitude of the flash-induced ΔA_520 nm_. The slope of the linear portion was taken as the rate constant of ATP synthase in the dark (*g*_H_^+^_d_), and the x-intercept (ΔA_520 nm_ amplitude) was taken as *pmf* threshold of activation (*pmf*_t_). For the overnight dark adaptation, plants were measured for all FIRK parameters at least 4 h after the growth chamber lights had turned off.

All amplitude-dependent ECS (ΔA_520 nm_) parameters were normalized to chlorophyll concentration of the leaf area measured.

### Fluorescence imaging acquisition and analysis

Tobacco cultivars “Petit Havana” and “SNN” were grown in a growth cabinet outfitted with a dynamic environmental photosynthetic imaging system [DEPI, (Cruz et al., 2016)] at 100 μmoles photons m^−2^ s^−1^ in a 16 h:8 h photoperiod until they were 5 weeks old. Plants were imaged in a 5-day experiment as described in (Cruz et al., 2016). To manage leaf movement in, raw images were initially processed in Visual Phenomics (Cruz et al., 2016), and then leaves were manually tracked and the fluorescence values were extracted in ImageJ (Schneider et al., 2012). NPQ, *q*_E_, and ϕ_II_ were calculated as in (Strand et al., 2017) with the exception that the F_M_” value for the calculation of *q*_E_ was taken after a dark relaxation period of 2 min.

### Accession numbers

Sequence data from this article can be found in the GenBank/EMBL data libraries under accession numbers NC_001879.2 (*Nicotiana tabacum*, complete plastid genome) for *atpB*, *atpF*, and *psaB* and NM_001325914 for *ATPC1*.

## Supplementary Material

kiad013_Supplementary_Data

## Supplemental data

The following materials are available in the online version of this article.

**Supplemental Table S1**. Synthetic nucleotides used in this study.

**Supplemental Figure S1**. Sanger chromatograms of all lines from DNA of ∼20 pooled seedlings resistant to spectinomycin. Black boxes indicate codons harboring mutations.

**Supplemental Figure S2**. Complex assembly of the ATP synthase in the wild type (WT), the *aadA* control (Ctrl) and the atpB mutants S13D-11A, S8A + S13D-23B, and S8D + S13D-12B.

**Supplemental Figure S3**. Nonphotochemical chlorophyll fluorescence quenching (NPQ) in *atpB* mutants during a 5 day DEPI experiment.

**Supplemental Figure S4**. Quantum yield of photosystem II (ϕ_II_) in *atpB* mutants during a 5 day DEPI experiment.

**Supplemental Figure S5**. Excitation quenching of chlorophyll fluorescence (*q_E_*) in *atpB* mutants during a 5 day DEPI experiment.

**Supplemental Figure S6**. Photosynthetic parameters in the *ATPC1* antisense mutant during a 5 day DEPI experiment.

## Abbreviations

CBB: Coomassie Brilliant Blue
CF_o_: membrane fraction of ATP synthase
CF_1_: soluble fraction of ATP synthase
*pmf*: protonmotive force
q_E_: exciton quenching
NPQ: nonphotochemical quenching
ΔpH: fraction of *pmf* stored as pH
*bf*: cytochrome *b_6_f* complex
Q_o_: quinol oxidation site
WT: wild type
PCR: polymerase chain reaction
ATP: adenosine triphosphate
DNA: deoxyribonucleic acid
RNA: ribonucleic acid
ECS: electrochromic shift
ECS*_t_*: total electrochromic shift
τ_ECS_: lifetime of the electrochromic shift
ϕ_II_: quantum yield of photosystem II
*g*_H_^+^: transthylakoid proton conductivity
PSI: photosystem I
PSII: photosystem II
*g*_H_^+^_d_: transthylakoid proton conductivity in the dark
*pmf*_t_: protonmotive force threshold of activation
ON: overnight
CO_2_: carbon dioxide
NADPH: nicotinamide adenine dinucleotide phosphate
BN-PAGE: blue-native polyacrylamide gel electrophoresis

## References

[kiad013-B1] Agrawal S , Karcher D, Ruf S, Bock R (2020) The functions of chloroplast glutamyl-tRNA in translation and tetrapyrrole biosynthesis1[open]. Plant Physiol 183(1): 263–276 32071153 10.1104/pp.20.00009 PMC7210637

[kiad013-B2] Baker NR (2008) Chlorophyll fluorescence: a probe of photosynthesis in vivo. Annu Rev Plant Biol 59(1): 89–113 18444897 10.1146/annurev.arplant.59.032607.092759

[kiad013-B3] Baker NR , Harbinson J, Kramer DM (2007) Determining the limitations and regulation of photosynthetic energy transduction in leaves. Plant Cell Environ 30(9): 1107–1125 17661750 10.1111/j.1365-3040.2007.01680.x

[kiad013-B4] Bock R (2001) Transgenic plastids in basic research and plant biotechnology. J Mol Biol 312(3): 425–438 11563907 10.1006/jmbi.2001.4960

[kiad013-B5] Bock R (2015) Engineering plastid genomes: methods, tools, and applications in basic research and biotechnology. Annu Rev Plant Biol 66(1): 211–241 25494465 10.1146/annurev-arplant-050213-040212

[kiad013-B6] Bunney TD , Van Walraven HS, De Boer AH (2001) 14-3-3 Protein is a regulator of the mitochondrial and chloroplast ATP synthase. Proc Natl Acad Sci U S A 98(7): 4249–4254 11274449 10.1073/pnas.061437498 PMC31211

[kiad013-B7] Caroca R , Howell KA, Malinova I, Burgos A, Tiller N, Pellizzer T, Annunziata MG, Hasse C, Ruf S, Karcher D, et al (2021) Knockdown of the plastid-encoded acetyl-CoA carboxylase gene uncovers functions in metabolism and development. Plant Physiol 185(3): 1091–1110 33793919 10.1093/plphys/kiaa106 PMC8133629

[kiad013-B8] Cruz JA , Savage LJ, Zegarac R, Hall CC, Satoh-Cruz M, Davis GA, Kovac WK, Chen J, Kramer DM (2016) Dynamic environmental photosynthetic imaging reveals emergent phenotypes. Cell Syst 2(6): 365–377 27336966 10.1016/j.cels.2016.06.001

[kiad013-B9] Davis GA , Kanazawa A, Schöttler MA, Kohzuma K, Froehlich JE, William Rutherford A, Satoh-Cruz M, Minhas D, Tietz S, Dhingra A, et al (2016) Limitations to photosynthesis by proton motive force-induced photosystem II photodamage. Elife 5: e16921 27697149 10.7554/eLife.16921 PMC5050024

[kiad013-B10] del Riego G , Casano LM, Martín M, Sabater B (2006) Multiple phosphorylation sites in the beta subunit of thylakoid ATP synthase. Photosynth Res 89(1): 11–18 16832703 10.1007/s11120-006-9078-4

[kiad013-B11] Doyle JJ , Doyle JL (1987) A rapid DNA isolation procedure for small quantities of fresh leaf tissue. Phytochem Bull 19(1): 11–15

[kiad013-B12] Eberhard S , Finazzi G, Wollman F-A (2008) The dynamics of photosynthesis. Annu Rev Genet 42(1): 463–515 18983262 10.1146/annurev.genet.42.110807.091452

[kiad013-B13] Fuentes I , Karcher D, Bock R (2012) Experimental reconstruction of the functional transfer of intron-containing plastid genes to the nucleus. Curr Biol 22(9): 763–771 22503505 10.1016/j.cub.2012.03.005

[kiad013-B14] Gibson DG (2011) Enzymatic assembly of overlapping DNA fragments. Methods Enzymol 498: 349–361 21601685 10.1016/B978-0-12-385120-8.00015-2 PMC7149801

[kiad013-B15] Hahn A , Vonck J, Mills DJ, Meier T, Kühlbrandt W (2018) Structure, mechanism, and regulation of the chloroplast ATP synthase. Science 360(6389): eaat4318 29748256 10.1126/science.aat4318 PMC7116070

[kiad013-B16] Hall CC , Cruz J, Wood M, Zegarac R, DeMars D, Carpenter J, Kanazawa A, Kramer D (2013) Photosynthetic measurements with the idea spec: an integrated diode emitter array spectrophotometer/fluorometer. Photosynth. Res. Food, fuel futur. *In*15th International Conference on Photosynthesis. Springer, Berlin Heidelberg, pp 184–188

[kiad013-B17] Järvi S , Suorsa M, Paakkarinen V, Aro EM (2011) Optimized native gel systems for separation of thylakoid protein complexes: novel super- and mega-complexes. Biochem J 439(2): 207–214 21707535 10.1042/BJ20102155

[kiad013-B18] Kanazawa A , Kramer DM (2002) In vivo modulation of nonphotochemical exciton quenching (NPQ) by regulation of the chloroplast ATP synthase. Proc Natl Acad Sci U S A 99(20): 12789–12794 12192092 10.1073/pnas.182427499 PMC130538

[kiad013-B19] Kanekatsu M , Saito H, Motohashi K, Hisabori T (1998) The beta subunit of chloroplast ATP synthase (CFoCF1-ATPase) is phosphorylated by casein kinase II. Biochem Mol Biol Int 46(1): 99–105 9784844 10.1080/15216549800203602

[kiad013-B20] Kohzuma K , Cruz JA, Akashi K, Hoshiyasu S, Munekage YN, Yokota A, Kramer DM (2009) The long-term responses of the photosynthetic proton circuit to drought. Plant Cell Environ 32(3): 209–219 19021886 10.1111/j.1365-3040.2008.01912.x

[kiad013-B21] Kohzuma K , Dal Bosco C, Kanazawa A, Dhingra A, Nitschke W, Meurer J, Kramer DM (2012) Thioredoxin-insensitive plastid ATP synthase that performs moonlighting functions. Proc Natl Acad Sci U S A 109(9): 3293–3298 22328157 10.1073/pnas.1115728109 PMC3295299

[kiad013-B22] Kohzuma K , Dal Bosco C, Meurer J, Kramer DM (2013) Light- and metabolism-related regulation of the chloroplast ATP synthase has distinct mechanisms and functions. J Biol Chem 288(18): 13156–13163 23486473 10.1074/jbc.M113.453225 PMC3642356

[kiad013-B23] Kramer DM , Avenson TJ, Edwards GE (2004) Dynamic flexibility in the light reactions of photosynthesis governed by both electron and proton transfer reactions. Trends Plant Sci 9(7): 349–357 15231280 10.1016/j.tplants.2004.05.001

[kiad013-B24] Kramer DM , Crofts AR (1989) Activation of the chloroplast ATPase measured by the electrochromic change in leaves of intact plants. Biochim Biophys Acta - Bioenerg 976(1): 28–41

[kiad013-B25] Kramer DM , Wise RR, Frederick JR, Alm DM, Hesketh JD, Ort DR, Crofts AR (1990) Regulation of coupling factor in field-grown sunflower: a Redox model relating coupling factor activity to the activities of other thioredoxin-dependent chloroplast enzymes. Photosynth Res 26(3): 213–222 24420586 10.1007/BF00033134

[kiad013-B26] Krech K , Fu HY, Thiele W, Ruf S, Schöttler MA, Bock R (2013) Reverse genetics in complex multigene operons by co-transformation of the plastid genome and its application to the open Reading frame previously designated psbN. Plant J 75(6): 1062–1074 23738654 10.1111/tpj.12256

[kiad013-B27] Li X-P , Muller-Moule P, Gilmore AM, Niyogi KK (2002) PsbS-dependent enhancement of feedback de-excitation protects photosystem II from photoinhibition. Proc Natl Acad Sci U S A 99(23): 15222–7 12417767 10.1073/pnas.232447699 PMC137571

[kiad013-B28] Moreno JC , Martínez-Jaime S, Schwartzmann J, Karcher D, Tillich M, Graf A, Bock R (2018) Temporal proteomics of inducible RNAi lines of Clp protease subunits identifies putative protease substrates. Plant Physiol 176(2): 1485–1508 29229697 10.1104/pp.17.01635 PMC5813558

[kiad013-B29] Ort DR , Merchant SS, Alric J, Barkan A, Blankenship RE, Bock R, Croce R, Hanson MR, Hibberd JM, Long SP, et al (2015) Redesigning photosynthesis to sustainably meet global food and bioenergy demand. Proc Natl Acad Sci U S A 112(28): 8529–8536 26124102 10.1073/pnas.1424031112 PMC4507207

[kiad013-B30] Reiland S , Messerli G, Baerenfaller K, Gerrits B, Endler A, Grossmann J, Gruissem W, Baginsky S (2009) Large-scale Arabidopsis phosphoproteome profiling reveals novel chloroplast kinase substrates and phosphorylation networks. Plant Physiol 150(2): 889–903 19376835 10.1104/pp.109.138677 PMC2689975

[kiad013-B31] Rott M , Martins NF, Thiele W, Lein W, Bock R, Kramer DM, Schöttler MA (2011) ATP Synthase repression in tobacco restricts photosynthetic electron transport, CO_2_ assimilation, and plant growth by overacidification of the thylakoid lumen. Plant Cell 23(1): 304–321 21278125 10.1105/tpc.110.079111 PMC3051256

[kiad013-B32] Rühle T , Razeghi JA, Vamvaka E, Viola S, Gandini C, Kleine T, Schünemann D, Barbato R, Jahns P, Leister D (2014) The Arabidopsis protein CONSERVED ONLY IN THE GREEN LINEAGE160 promotes the assembly of the membranous part of the chloroplast ATP synthase. Plant Physiol 165(1): 207–226 24664203 10.1104/pp.114.237883 PMC4012581

[kiad013-B33] Sandoval-Ibáñez O , Rolo D, Ghandour R, Hertle AP, Armarego-Marriott T, Sampathkumar A, Zoschke R, Bock R (2022) De-etiolation-induced protein 1 (DEIP1) mediates assembly of the cytochrome b 6 f complex in Arabidopsis. Nat Commun 13(1): 1–16 35831297 10.1038/s41467-022-31758-7 PMC9279372

[kiad013-B34] Schägger H (2006) Tricine-SDS-PAGE. Nat Protoc 1(1): 16–22 17406207 10.1038/nprot.2006.4

[kiad013-B35] Schneider CA , Rasband WS, Eliceiri KW (2012) NIH Image to ImageJ: 25 years of image analysis. Nat Methods 9(7): 671–675 22930834 10.1038/nmeth.2089 PMC5554542

[kiad013-B36] Schöttler MA , Tóth SZ, Boulouis A, Kahlau S (2015) Photosynthetic complex stoichiometry dynamics in higher plants: biogenesis, function, and turnover of ATP synthase and the cytochrome b6f complex. J Exp Bot 66(9): 2373–2400 25540437 10.1093/jxb/eru495

[kiad013-B37] Strand DD , Fisher N, Davis GA, Kramer DM (2016) Redox regulation of the antimycin A sensitive pathway of cyclic electron flow around photosystem I in higher plant thylakoids. Biochim Biophys Acta 1857(1): 1–6 26235611 10.1016/j.bbabio.2015.07.012

[kiad013-B38] Strand DD , Livingston AK, Satoh-Cruz M, Koepke T, Enlow HM, Fisher N, Froehlich JE, Cruz JA, Minhas D, Hixson KK, et al (2017) Defects in the expression of chloroplast proteins leads to H_2_O_2_ accumulation and activation of cyclic electron flow around photosystem I. Front Plant Sci 7: 2073 28133462 10.3389/fpls.2016.02073 PMC5233679

[kiad013-B39] Suorsa M , Järvi S, Grieco M, Nurmi M, Pietrzykowska M, Rantala M, Kangasjärvi S, Paakkarinen V, Tikkanen M, Jansson S, et al (2012) PROTON GRADIENT REGULATION5 is essential for proper acclimation of Arabidopsis photosystem I to naturally and artificially fluctuating light conditions. Plant Cell 24(7): 2934–2948 22822205 10.1105/tpc.112.097162 PMC3426124

[kiad013-B40] Svab Z , Maliga P (1993) High-frequency plastid transformation in tobacco. Proc Natl Acad Sci U S A 90(3):913–917 8381537 10.1073/pnas.90.3.913 PMC45780

[kiad013-B41] Takizawa K , Kanazawa A, Kramer DM (2008) Depletion of stromal Pi induces high “energy-dependent” antenna exciton quenching (qE) by decreasing proton conductivity at CFO-CF1 ATP synthase. Plant Cell Environ 31(2): 235–243 17996016 10.1111/j.1365-3040.2007.01753.x

[kiad013-B42] Vilela B , Pagès M, Riera M (2015) Emerging roles of protein kinase CK_2_ in abscisic acid signaling. Front Plant Sci 6: 966 26579189 10.3389/fpls.2015.00966 PMC4630567

[kiad013-B43] Wu G , Ortiz-Flores G, Ortiz-Lopez A, Ort DR (2007) A point mutation in atpC1 raises the redox potential of the Arabidopsis chloroplast ATP synthase gamma-subunit regulatory disulfide above the range of thioredoxin modulation. J Biol Chem 282(51): 36782–9 17959606 10.1074/jbc.M707007200

[kiad013-B44] Zhang R , Cruz JA, Kramer DM, Magallanes-Lundback ME, Dellapenna D, Sharkey TD (2009) Moderate heat stress reduces the pH component of the transthylakoid proton motive force in light-adapted, intact tobacco leaves. Plant Cell Environ 32(11): 1538–1547 19558623 10.1111/j.1365-3040.2009.02018.x

