# Supplementary material for: Characterization of mutants deficient in N-terminal phosphorylation of the chloroplast ATP synthase subunit β
Source: Plant Physiol. 2023 Jan 13;191(3):1818–35. doi: 10.1093/plphys/kiad013 (PMC10022623; doi:10.1093/plphys/kiad013)
Supplement: kiad013_Supplementary_Data [file kiad013_supplementary_data.pdf]

Name	Sequence (5'--> 3')	Use
P1	tcccacgtgcccgggcgtttCTAAATGTGCAAATGTCGTAGC	PCR amplification for vector construction
P2	tatagggaatttaaatttaatCCCTGCTTCTTCAGGTGG	PCR amplification for vector construction
P3	agcgtggaaaccccagaaccagcagtagtaggattgattctca	PCR amplification for mutagenesis
P4	tgagaatcaatcctactactgctggttctggggtttccacgct	PCR amplification for mutagenesis
P5	gggttttttttttcaagcgtagcaaccccagaaccagaagtag	PCR amplification for mutagenesis
P5	ctacttctggttctggggttgctacgcttgaaaaaaaaaaccc	PCR amplification for mutagenesis
P7	agcgtggaaaccccagaaccatcagtagtaggattgattctca	PCR amplification for mutagenesis
P8	tgagaatcaatcctactactgatggttctggggtttccacgct	PCR amplification for mutagenesis
P9	gggttttttttttcaagcgtatcaaccccagaaccagaagtag	PCR amplification for mutagenesis
P10	ctacttctggttctggggttgatacgcttgaaaaaaaaaaccc	PCR amplification for mutagenesis
P11	gggttttttttttcaagcgtagcaaccccagaaccagcagtag	PCR amplification for mutagenesis
P12	ctactgctggttctggggttgctacgcttgaaaaaaaaaaccc	PCR amplification for mutagenesis
P13	gggttttttttttcaagcgtatcaaccccagaaccatcagtag	PCR amplification for mutagenesis
P14	ctactgatggttctggggttgatacgcttgaaaaaaaaaaccc	PCR amplification for mutagenesis
P15	gggttttttttttcaagcgtatcaaccccagaaccagcagtag	PCR amplification for mutagenesis
P16	ctactgctggttctggggttgatacgcttgaaaaaaaaaaccc	PCR amplification for mutagenesis
P17	atgagaatcaatcctactactgAtggttctggggttgcTacgcttgaaaa	PCR amplification for vector construction
P18	ttttcaagcgtAgcaaccccagaaccaTcagtagtaggattgattctcat	PCR amplification for vector construction
P19	CCCAGAAAGAGGCTGGCCC	PCR for Southern blot probe
P20	CCCAAGGGGCGGGAACTGC	PCR for Southern blot probe
P21	CTCTGGTAGTTCAAGGTCGAG	PCR for Northern blot probe
P22	GTATCCAACTGTATAAAGGCGG	PCR for Northern blot probe

Supplemental Table S1. Synthetic nucleotides used in this study.

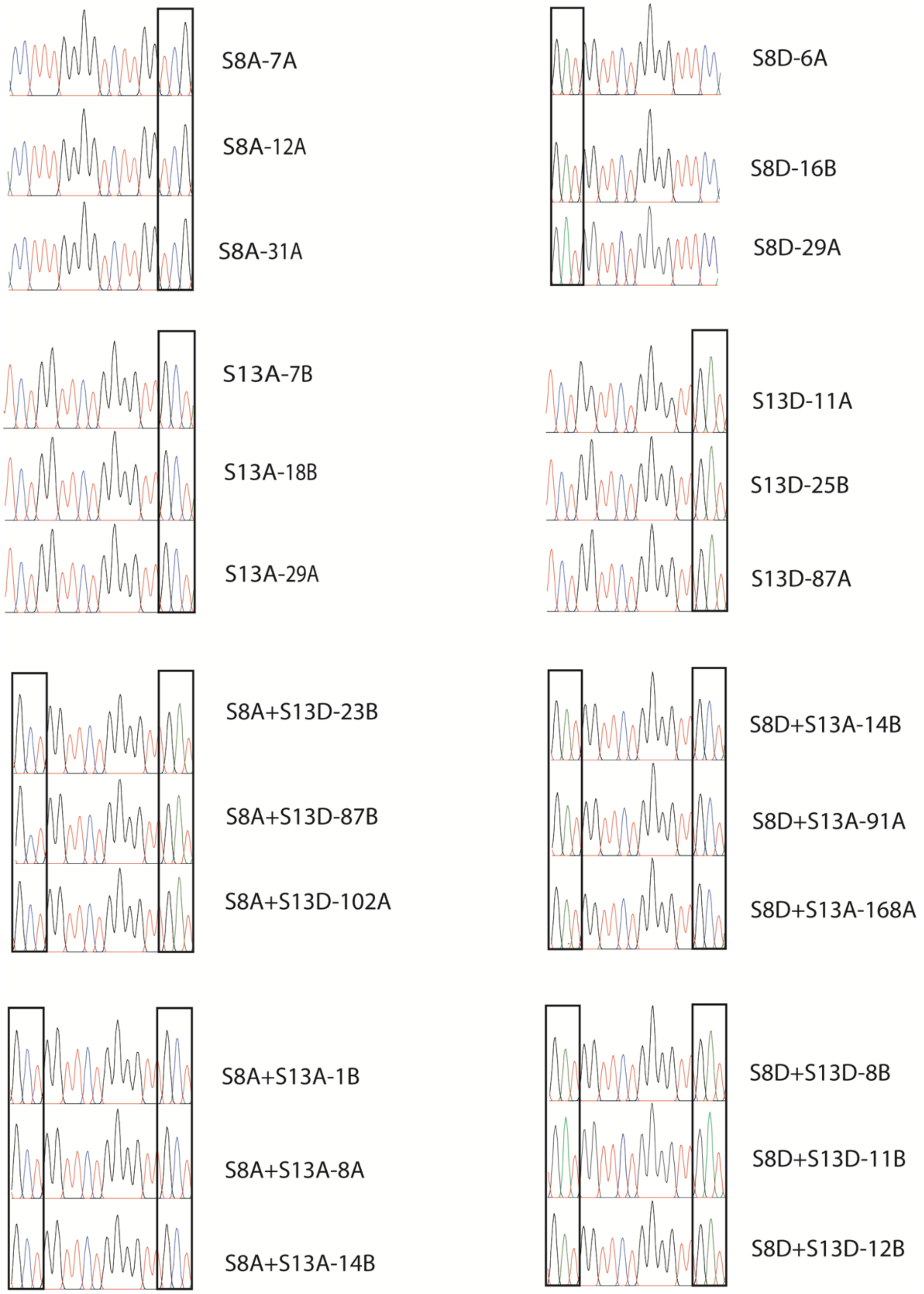

Supplemental Figure S1. Sanger chromatograms of all lines from DNA of ~20 pooled seedlings resistant to spectinomycin. Black boxes indicate codons harboring mutations.

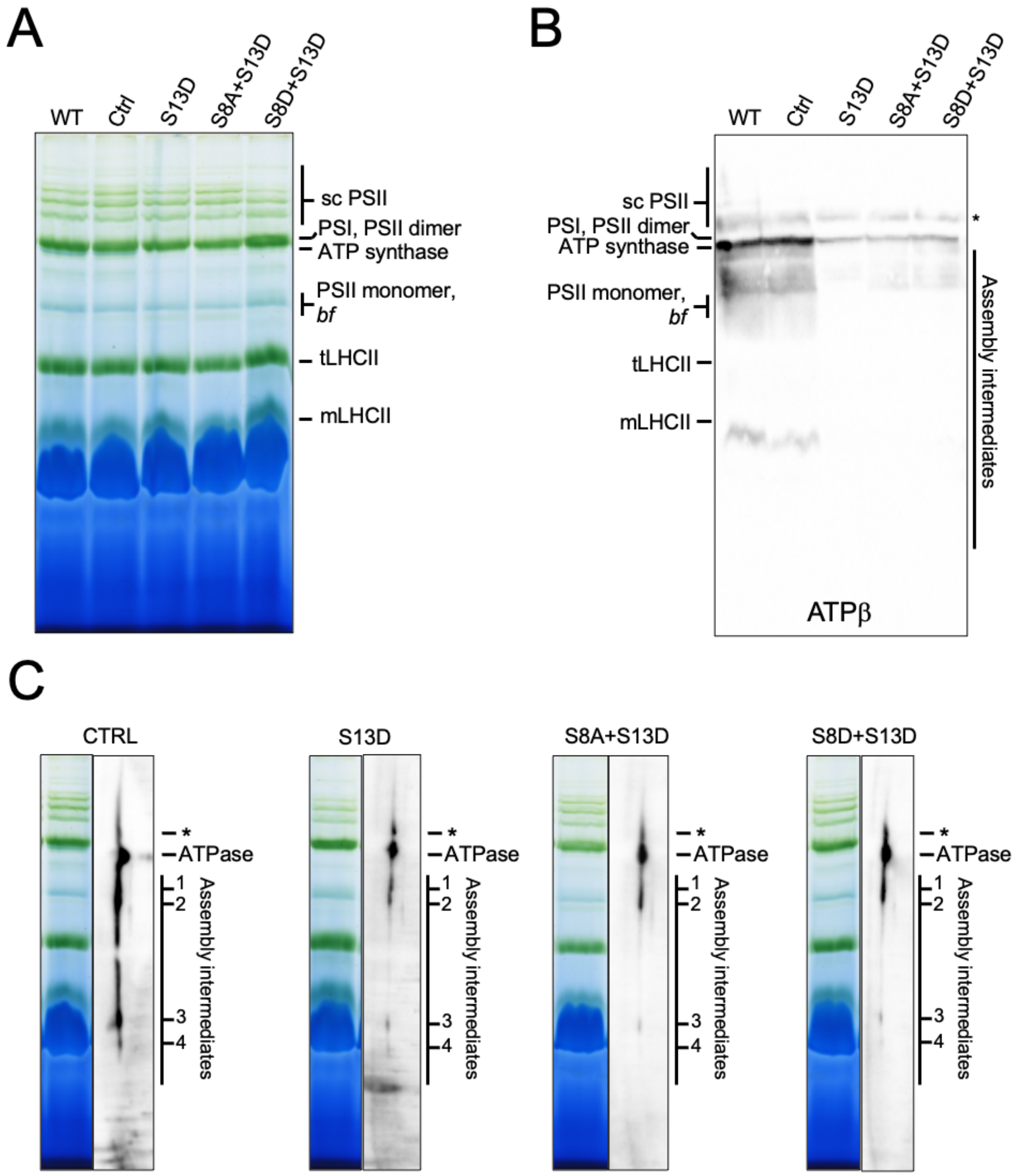

Supplemental Figure S2. Complex assembly of the ATP synthase in the wild type (WT), the *aadA* control (Ctrl) and the *atpB* mutants S13D-11A, S8A+S13D-23B, and S8D+S13D-12B. (A) BN-PAGE of wild type (WT), control (Ctrl) and the *atpB* mutants S13D, S8A+S13D and S8D+S13D. Thylakoid samples equal to 20μg chlorophyll were solubilized in β-DDM and resolved in a 8-13.9% (w/v) native gradient gel. The labels at the right indicate the PSII supercomplexes (scPSII), photosystem I (PSI), photosystem II dimer (PSII dimer), the ATP synthase, photosystem II monomer (PSII monomer), cytochrome b_6f (*bf*) and the trimer (tLHCII) and monomeric (mLHCII) light harvesting complex II. (B) Immunoblot of the first dimensional gel. The anti-ATPβ antibody was employed for immunodetection and the labels at the left indicate the complexes as in (A). The labels at the left indicate the high molecular weight complex. (C) Immunoblot of second dimension SDS PAGE gel using the anti-ATPβ antibody.

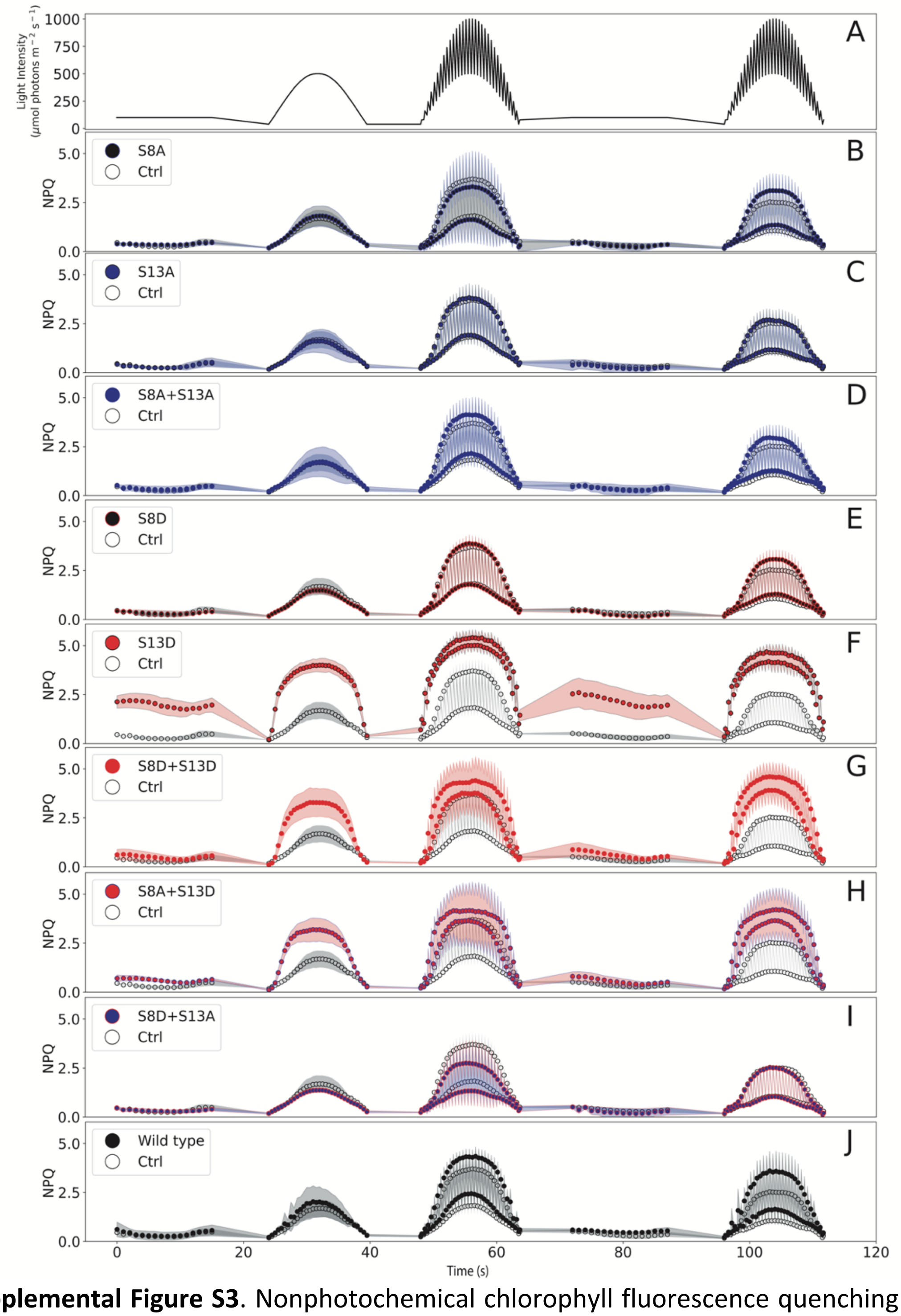

Supplemental Figure S3. Nonphotochemical chlorophyll fluorescence quenching (NPQ) in *atpB* mutants during a 5 day DEPI experiment. (A) Light intensity during the course of the experiment. (B-J) NPQ in representative *atpB* mutant lines. Data represents mean +/- SD, n = 3. Lines used: *N. tabacum* 'Petit Havana' (WT), Ctrl (*aadA* control line), S8A-7A, S13A-18B, S8A+S13A-8A, S8D-29A, S13D-25B, S8D+S13D-12B, S8A+S13D-102A and S8D+S13A-91A.

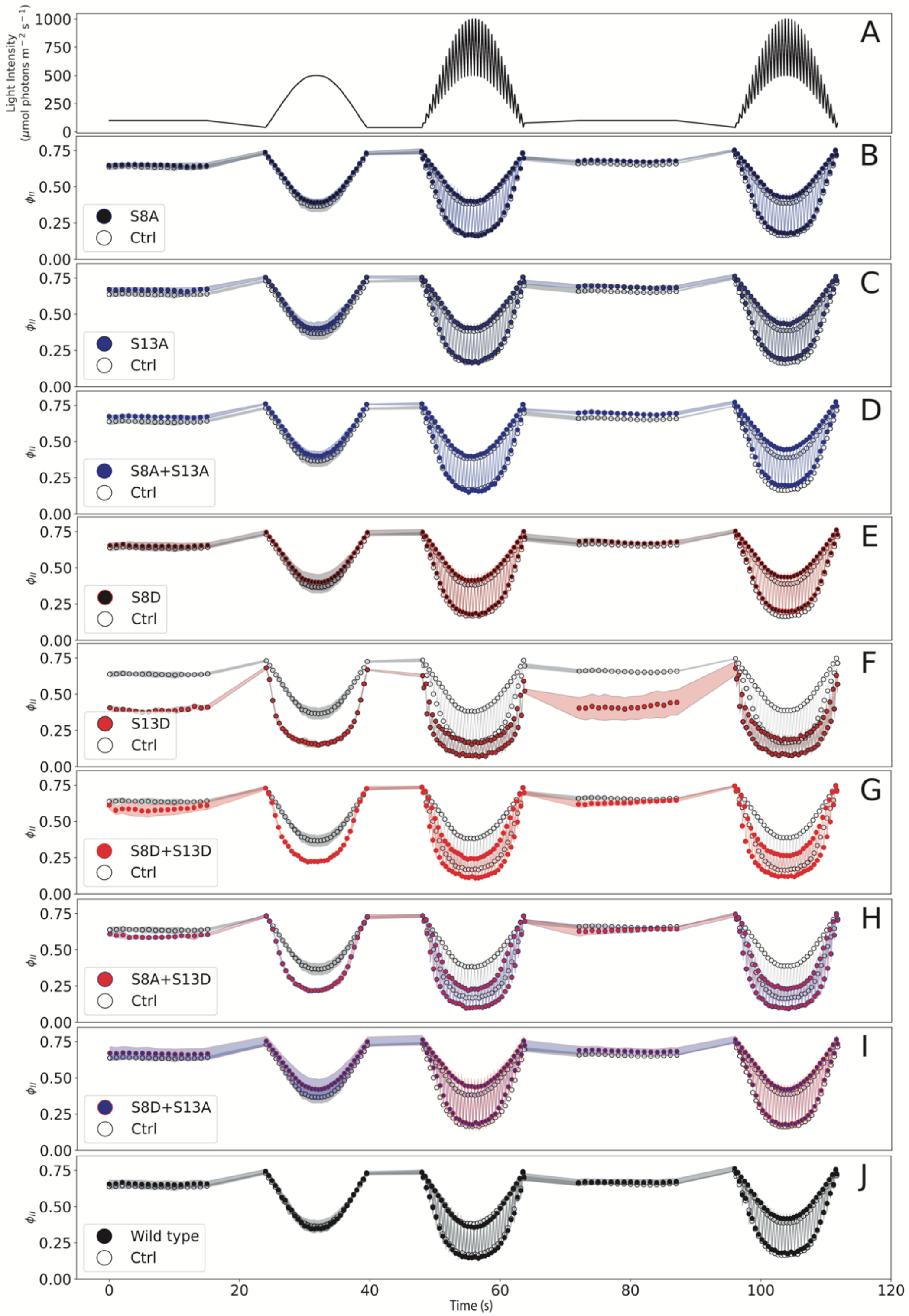

Supplemental Figure S4. Quantum yield of photosystem II (ϕ_{II}) in *atpB* mutants during a 5 day DEPI experiment. (A) Light intensity during the course of the experiment. (B-J) ϕ_{II} in representative atpB mutant lines. Data represents mean +/- SD, n = 3. Lines used: *N. tabacum* 'Petit Havana' (WT), Ctrl (*aadA* control line), S8A-7A, S13A-18B, S8A+S13A-8A, S8D-29A, S13D-25B, S8D+S13D-12B, S8A+S13D-102A and S8D+S13A-91A.

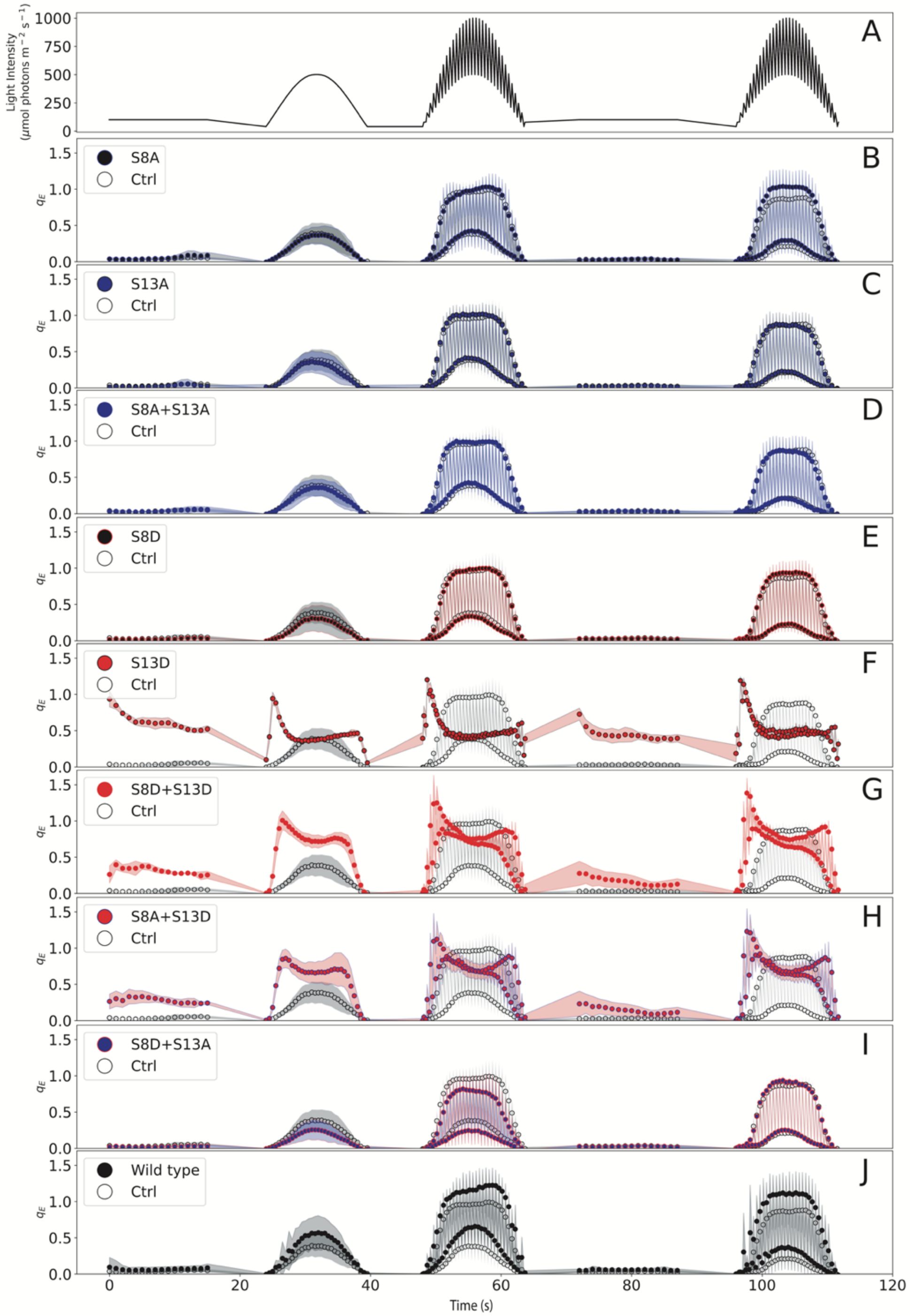

Supplemental Figure S5. Excitation quenching of chlorophyll fluorescence (q_E) in *atpB* mutants during a 5 day DEPI experiment. (A) Light intensity during the course of the experiment. (B-J) q_E in representative *atpB* mutant lines. Data represents mean +/- SD, n = 3. Lines used: *N. tabacum* 'Petit Havana' (WT), Ctrl (*aadA* control line), S8A-7A, S13A-18B, S8A+S13A-8A, S8D-29A, S13D-25B, S8D+S13D-12B, S8A+S13D-102A and S8D+S13A-91A.

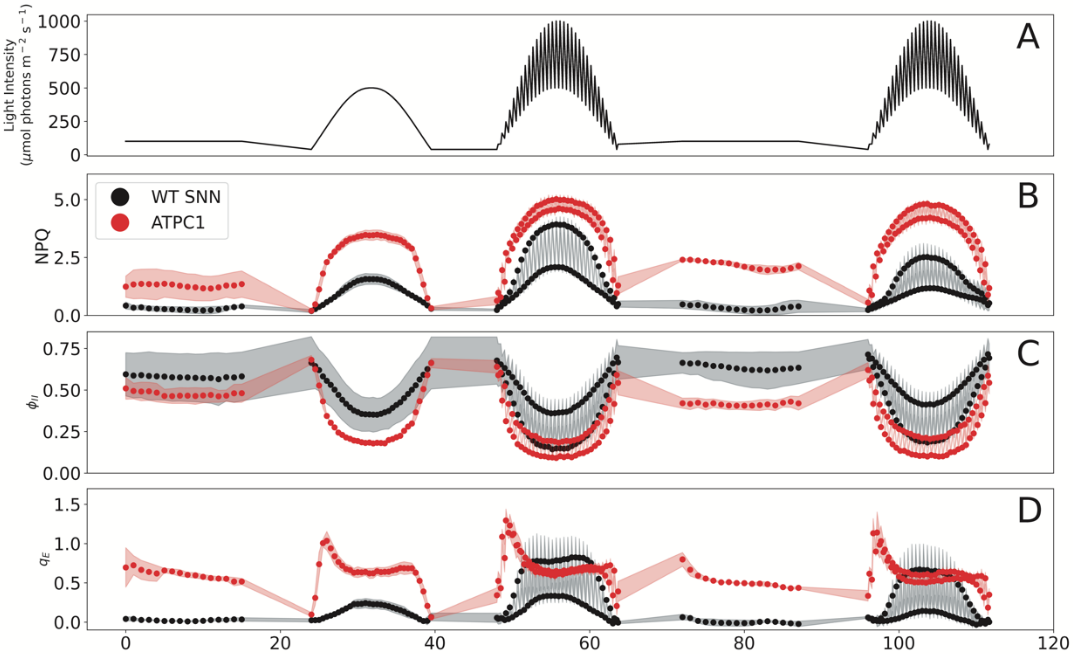

Supplemental Figure S6. Photosynthetic parameters in the *ATPC1* antisense mutant during a 5 day DEPI experiment. (A) Light intensity during the course of the experiment. (B) Nonphotochemical chlorophyll fluorescence quenching (NPQ); (C) Quantum yield of photosystem II (ϕ_{II}); (D) Excitation quenching of chlorophyll fluorescence (q_E). Data represents mean +/- SD, n = 3. Lines used: *N. tabacum* ' Samsun-NN' (WT SNN) and *ATPC1* antisense mutant (ATPC1).
